# Enthesitis on Chip – A Model for Studying Acute and Chronic Inflammation of the Enthesis and its Pharmacological Treatment

**DOI:** 10.1002/adhm.202401815

**Published:** 2024-08-27

**Authors:** Francesca Giacomini, Hoon Suk Rho, Maria Eischen‐Loges, Zeinab Tahmasebi Birgani, Clemens van Blitterswijk, Martijn van Griensven, Stefan Giselbrecht, Pamela Habibović, Roman Truckenmüller

**Affiliations:** ^1^ Department of Instructive Biomaterials Engineering MERLN Institute for Technology‐Inspired Regenerative Medicine Maastricht University Universiteitssingel 40 Maastricht 6229 ER The Netherlands; ^2^ Department of Cell Biology‐Inspired Tissue Engineering MERLN Institute for Technology‐Inspired Regenerative Medicine Maastricht University Universiteitssingel 40 Maastricht 6229 ER The Netherlands

**Keywords:** drug testing, enthesis, enthesitis, inflammation, organ on chip

## Abstract

Enthesitis, the inflammation of the enthesis, which is the point of attachment of tendons and ligaments to bones, is a common musculoskeletal disease. The inflammation often originates from the fibrocartilage region of the enthesis as a consequence of mechanical overuse or ‐load and consequently tissue damage. During enthesitis, waves of inflammatory cytokines propagate in(to) the fibrocartilage, resulting in detrimental, heterotopic bone formation. Understanding of human enthesitis and its treatment options is limited, also because of lacking in vitro model systems that can closely mimic the pathophysiology of the enthesis and can be used to develop therapies. In this study, an enthes(it)is‐on‐chip model is developed. On opposite sides of a porous culture membrane separating the chip's two microfluidic compartments, human mesenchymal stromal cells are selectively differentiated into tenocytes and fibrochondrocytes. By introducing an inflammatory cytokine cocktail into the fibrochondrocyte compartment, key aspects of acute and chronic enthesitis, measured as increased expression of inflammatory markers, can be recapitulated. Upon inducing chronic inflammatory conditions, hydroxyapatite deposition, enhanced osteogenic marker expression and reduced secretion of tissue‐related extracellular matrix components are observed. Adding the anti‐inflammatory drug celecoxib to the fibrochondrocyte compartment mitigates the inflammatory state, demonstrating the potential of the enthesitis‐on‐chip model for drug testing.

## Graphical abstract

Enthesitis, the inflammation of the enthesis, which is the point of attachment of tendons and ligaments to bones, is a common musculoskeletal disease. In this study, an enthes(it)is‐on‐chip model is developed. Using inflammatory cytokines, key aspects of acute and chronic enthesitis can be recapitulated. Adding an anti‐inflammatory drug mitigates the inflammation, demonstrating the potential of the model for drug testing.

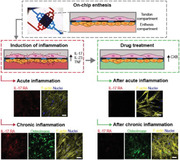

## 1 Introduction

The site of insertion of tendons into bone, or enthesis, is a specialized interface tissue transmitting mechanical forces from muscles to the skeletal system.^[^ ^1^ ^]^ These functions are facilitated by the unique architecture of the enthesis, characterized by the gradual transition of cell types and extracellular matrix (ECM) composition and structure from tendon to bone tissue.^[^ ^2^ ^]^

Enthesitis is a common debilitating musculoskeletal disease characterized by progressive inflammation and remodeling of the enthesis, resulting in tissue degeneration.^[^ ^3^ ^]^ Acute enthesitis often results from repeated mechanical overloading during sports activities. Clinical examples of acute enthesitis, such as “tennis elbow” and “golfer elbow”, generally involve single entheses and can propagate to the body of the tendon.^[^ ^4^ ^]^ On the other hand, chronic enthesitis is mainly a feature of spondyloarthritis (SpA) or psoriatic arthritis (PsA) patients and occurs in response to low‐level mechanical strains.^[^ ^3^ ^]^ Here, the inflammation process can target multiple entheses and translates into severe pain and bone enlargement.^[^ ^5^ ^]^

While only few studies addressed enthesitis treatment,^[^ ^6^ ^]^ more effort went into characterizing the initiation and progression of the disease. For example, mechanical stress has been identified as a major factor for inducing Achilles tendon enthesitis in rats^[^ ^7^ ^]^ and mice,^[^ ^8^ ^]^ which stimulates a signaling cascade resulting in the activation of the interleukin (IL‐)23‐IL‐17 pathway.

IL‐23 is a cytokine derived from macrophages and dendritic cells that is sufficient to activate enthesitis in vivo independently from mechanical overload.^[^ ^9^ ^]^ IL‐23 targeting resident entheseal cells triggers the production of the inflammatory cytokines Tumor Necrosis Factor (TNF‐)*α* and IL‐17, which are causing the establishment and maintenance of the inflammatory state.^[^ ^10^ ^]^ In detail, TNF‐*α* overexpression targeting resident mesenchymal cells and fibroblasts in the enthesis is sufficient to establish and sustain enthesitis.^[^ ^11^ ^]^ IL‐17 is a key amplifier of the inflammatory response, promoting neutrophil migration and activation and, in combination with TNF‐*α*, stimulating the release of a variety of pro‐inflammatory cytokines (including IL‐6 and IL‐8) and mediators from resident mesenchymal cells.^[^ ^9^ ^]^ As a consequence, chronic enthesitis typically results in new bone formation at the fibrocartilage region of the enthesis, as demonstrated in human tissues.^[^ ^12^ ^]^ This process involves also IL‐17 and IL‐22, and is initiated by resident mesenchymal cells, which have proliferative potential and further differentiate into osteoblasts.^[^ ^13^ ^]^

Because of the limited accessibility of human tissue, the current knowledge about enthesitis was mainly generated from studies in mice.^[^ ^8^, ^9^, ^13^, ^14^ ^]^ Yet, this system only insufficiently recapitulates human enthesitis, for example, in terms of size and mechanical loading and is restrained by maintenance costs and growing ethical concerns.^[^ ^15^ ^]^ Therefore, the development of new enthesitis in vitro models from human‐derived cells might provide a more valid and sustainable alternative.

In the last years, on‐chip models have been used to recreate several tissue or organs and their pathophysiological conditions,^[^ ^16^ ^]^ such as for lungs,^[^ ^17^ ^]^ kidneys,^[^ ^18^ ^]^ intestine,^[^ ^19^ ^]^ heart,^[^ ^20^ ^]^ and liver^[^ ^21^ ^]^ and for musculoskeletal tissues and related inflammatory disorders.^[^ ^22^ ^]^ Organ‐on‐chip (OoC) technologies successfully recapitulate physiologically relevant compartmentalized microenvironments, including tissue‐specific cell types and ECM components. In addition, on‐chip systems allow the incorporation of dynamic elements by exploiting continuous perfusion with nutrients or soluble molecules and controlled application of biochemical, mechanical or electrical stimuli.^[^ ^23^ ^]^ For instance, Lin et al. developed a two‐chamber device modeling a chondral and an osseous compartment by controlling the exposure of human mesenchymal stromal cells (hMSCs) and induced pluripotent stem cells (iPSCs) to specific differentiation factors.^[^ ^24^ ^]^ This system allowed investigating osteochondral tissue physiology, the pathogenic mechanism of osteoarthritis (OA) upon introduction of IL‐1*β* in the medium flow and the protective effect of the anti‐inflammatory drug celecoxib (CXB).^[^ ^24b^ ^]^ Similarly, Oliveira et al. established a three‐dimensional (3D) inflammatory cartilage‐on‐chip model based on the co‐culture of primary human chondrocytes and the human monocyte cell line THP‐1, demonstrating the basic suitability of the system as a drug screening platform for OA.^[^ ^25^ ^]^ Moreover, several on‐chip models of rheumatoid arthritis (RA) have been established.^[^ ^26^ ^]^ Lyu et al. demonstrated the co‐culture of rat bone marrow‐derived and tendon stem cells seeded on decellularized rat tendon scaffolds in/under a growth factor concentration gradient generated by a microfluidic chip, to regulate stem cell activities for tendon‐to‐bone interface healing upon implantation into a rat model of tendon tear injury.^[^ ^27^ ^]^

**Figure 1:**
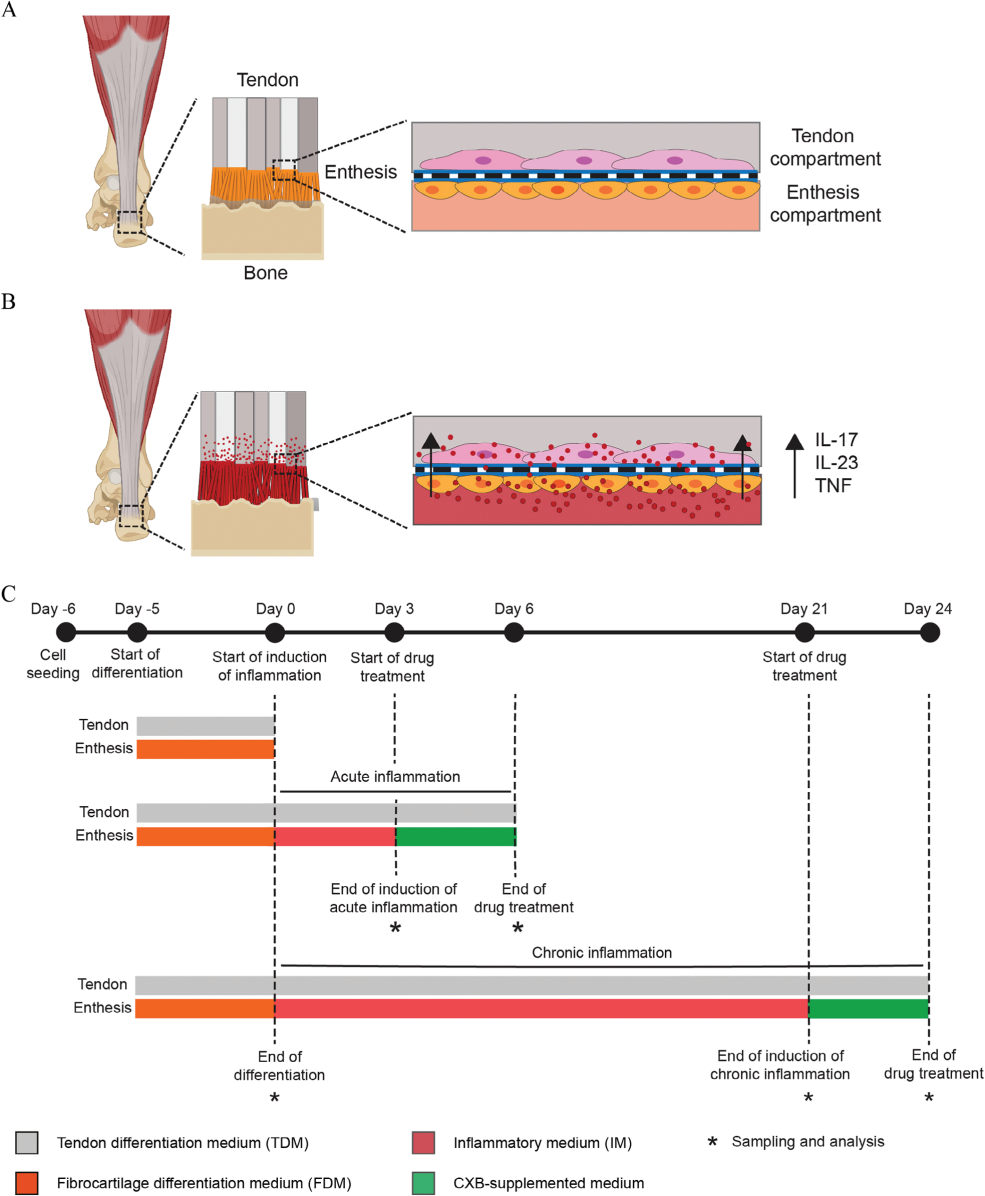
Concept of the enthesis‐on‐chip model. A) Entheses are the sites of insertion of tendons into bone. We conceptualized the enthesis organization in which a fibrocartilage layer is directly connected to the tendon as a simplified bilayer structure. Integrated in a microfluidic chip where a porous membrane separates a top compartment from a bottom one, the bilayer structure can be created by culturing tenocyte‐ and fibrochondrocyte‐like cells on the membrane's top and bottom side, respectively. B) As a consequence of tissue damage, the fibrocartilage region of the enthesis can be inflamed, leading to disruption of the original tissue architecture. The inflammatory response is characterized by the release of IL‐17, IL‐23 and TNF‐*α* mainly from leukocytes, which can propagate toward the tendon region. C) Overall experimental design to differentiate hMSCs into tenocytes and fibrochondrocytes in dedicated compartments of the chip, induce acute and chronic enthesitis and deliver CXB to mitigate the inflammation.

In this study, we developed a microfluidic chip from polydimethylsiloxane (PDMS) with two stacked compartments, which in the area of culture chambers were separated by a porous membrane from polycarbonate (PC) between them. The chip was designed to mimic the tendon region of the enthesis on one side of the membrane and the fibrocartilaginous region on the other side, together with corresponding cells and ECM (**Figure** **1**A). As the enthesis is a graded multi‐zone structure, a modeling of these two essential and central neighboring enthesis compartments using the mentioned classical and robust OoC design was considered an appropriate starting point for the, to the best of our knowledge, first study on the acute and chronic inflammation of the enthesis and its pharmacological treatment on a microfluidic chip. Apart from collagen coatings of the culture membrane “beneath” the cells, additional collagen overcasts of the two cell layers were applied to provide the cells with a more 3D‐like environment. The choice of a microfluidic implementation of the model was, in combination with the controlled, limited mass transport through the culture membrane, motivated by the continuous maintenance of the concentrations of the different (soluble) factors for differentiation in the culture media in the two compartments and by the constant exposure to factors for inflammation induction and mitigation throughout the whole related culture durations. The latter was conducted exclusively through the (lower) fibrocartilage compartment, which in vivo is next to the vascularized bone. The concentration maintenance and controlled permeability would not have been easy to implement in the static, non‐perfused setting of a culture insert or with a biofabricated (ECM) scaffold as cell support, respectively. After induction of selective differentiation of hMSCs into tenocyte‐ or fibrochondrocyte‐like cells in the separate compartments of the chip, we aimed at recapitulating enthesitis (Figure 1B). To mimic acute and chronic enthesitis, we exposed the cells in the fibrocartilaginous compartment to a combination of IL‐17, IL‐23 and TNF‐*α* for 3 or 21 days, respectively (Figure 1C). This exposure induced an increased expression of inflammatory markers and a concomitant release of inflammatory cytokines in the medium in both compartments. In chronic inflammatory conditions, we observed increased expression of osteogenic marker genes, and an alteration in the ECM composition, including hydroxyapatite (HA) deposition. Apart from inflammation, these are further clinical symptoms of enthesitis. Finally, we delivered CXB, a widely prescribed non‐steroidal anti‐inflammatory drug (NSAID), in the inflamed system and observed a mitigation of the inflammatory state (Figure 1C). This means that we could successfully recapitulate key aspects of the physiology and pathology of the tendon‐to‐fibrocartilage region of the enthesis. Findings from this study also confirm the functional crosstalk between tenocytes and fibrochondrocytes under inflamed conditions. The proposed or a similar platform might in future be used for drug screening, developing personalized treatments and investigating relevant signaling pathways involved in regulating the pathophysiology of the enthesis.

## 2 Results and Discussion

### 2.1 Design and Characterization of the Enthesis‐on‐Chip Device

**Figure 2:**
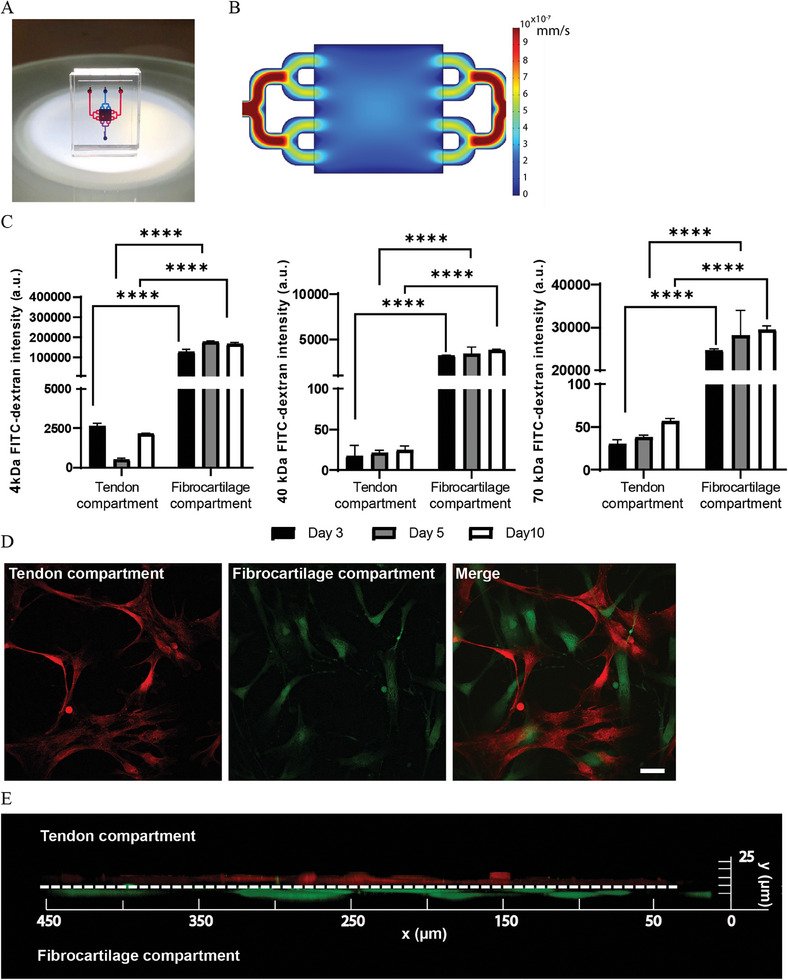
Design, microfabrication and characterization of the enthesis‐on‐chip device. A) Assembled microfluidic enthesis‐on‐chip device with the top compartment filled with red‐ and the bottom compartment with blue‐colored water. Length, width and height of the chip body as casted, assembled and cut are roughly 12, 8 and 4 mm, respectively. B) CFD simulation showing the computed distribution of the velocity of the laminar flow over the full area of one of the microfluidic culture chambers including parts of the branched inlet and outlet channels entering and exiting the chamber, respectively. The color legends at the right represent the local flow velocity in mm s^−1^. C) Quantification of the fluorescent intensity of FITC‐labeled dextrans with MWs of 4, 40 and 70 kDa in the tendon compartment at the top and fibrocartilage compartment at the bottom of the enthesis‐on‐chip device after 3, 5 and 10 days of perfusing the bottom compartment with the corresponding dextran solutions. Significance was determined by two‐way analysis of variance (ANOVA) followed by Tukey's post‐hoc test. ****p < 0.0001. N = 3. D) Confocal fluorescence (microscopy) images of hMSCs labeled with CellTracker Red in the tendon and CellTracker Green in the fibrocartilage compartment and cultured for 5 days under perfusion in the enthesis on chip. Scale bar represents 100 µm. E) 3D reconstruction from confocal fluorescence images as presented in (D), showing the cross section of the culture membrane in the microfluidic device with cells attached on opposite sides of the membrane. Scale bar represents 100 µm.

The enthesis on chip was developed first to generate and maintain a tendon‐to‐fibrocartilage interface‐like structure derived from hMSCs and second to mimic enthesitis. The design of the chip (**Figure** **2**A) is based on two distinct microfluidic compartments, one at the top for the tendon and one at the bottom for the fibrocartilage microenvironment. The two compartments included square chambers with a side length of 2.8 mm and a height of 75 µm, separated by a transparent ion track‐etched culture membrane from PC with a thickness of 10 µm. The chambers were on either side connected to inlet and outlet channels to allow for independent perfusion with medium and bioactive factors and for sampling. At the inlet and outlet side of the chambers, the channels were split in two steps to enable a more uniform flow profile in the chambers.

Each housing half of the microfluidic chip was fabricated from PDMS using a standard soft lithography process.^[^ ^28^ ^]^ The bonding of the housing halves and the enclosure of the membrane in between —the membrane had smaller side lengths than the chip housing— was conducted with PDMS mortar. This resulted in an irreversible, blockage‐free and leak‐tight assembly (Figure 2A).

We performed a computational fluid dynamics (CFD) simulation for the geometrically identical culture chambers of the chip to calculate the flow rate distribution inside the chambers (Figure 2B). The viscosity of the aqueous culture medium at 37 °C that was assumed to be sufficiently similar to that of water at the same temperature. The flow rate was set to 4 µL h^−1^, which corresponds to the common exchange of culture medium in the amount of the roughly 300 µL working volume of a well of a 96‐well plate every 3 days. The simulation showed an evenly distributed flow in the culture chamber. The maximum shear stress was calculated to be 0.01 dyn cm^−2^. This is well below values found to be necessary to promote hMSCs’ differentiation toward the osteogenic lineage by flow‐induced shear stress,^[^ ^29^ ^]^ while in this study differentiation was chosen to be controlled by differentiation medium/media only. The flow rate of 4 µL h^−1^ was applied for all microfluidic cell culture trials in this study.

We assessed the diffusion of small molecules across the thin porous culture membrane using solutions of fluorescein isothiocyanate (FITC)‐labeled dextran tracers of three different molecular weights (MWs). The bottom compartment of the microfluidic device was perfused with the dextran solutions while perfusing the top compartment with phosphate‐buffered saline (PBS) (Figure 2C). For this, a syringe pump with a multisyringe rack and two syringes mounted in it was used. The pump was set at the above‐introduced flow rate of 4 µL h^−1^. The MWs were chosen to be similar to those of the tenogenic and fibrochondrogenic inductive factors —the MW of Transforming Growth Factor (TGF‐)*β*2 and Growth/Differentiation Factor (GDF)5 is 25 and 27 kDa, respectively— and to the inflammatory factors – the MW of TNF‐*α*, IL‐17 and IL‐23 is 17, 31.3 and 53.5 kDa, respectively. The fluorescent intensities of the samples from the top and bottom compartments were measured after 3, 5 and 10 days of perfusion. For all three tracers, we observed significantly and considerably higher fluorescence levels in the bottom compartment of the chip relative to the top one, whereby the levels remained similar over the 10‐day period. This result demonstrates a controlled and comparatively small exchange of the tracers of all sizes through the culture membrane. The system therefore has the potential to generate two discrete microenvironments in terms of differentiation and inflammatory factors flown through the top and bottom compartments of the microfluidic device and at the same time allow cell‐cell communication through the pores in the membrane.

To promote cell attachment, both sides of the PC membrane were coated with a 0.5 mg mL^−1^ collagen type I solution. Then, hMSCs were seeded at a density of around 5000 cells cm^−2^ and allowed to adhere on both sides of the culture membrane. Finally, in both compartments, we added an additional thin collagen type I layer on the top of the cells to prevent their detachment during dynamic culturing and to provide them with a semi‐3D environment. To validate cell adhesion and growth on the membrane, we labeled the hMSCs with cell trackers. Under perfusion with basic medium (BM) over a period of up to 26 days, we observed successful attachment and proliferation of the cells on both the top and bottom side of the culture membrane, there uniformly populating the membrane's entire surface(s), showing an even cell distribution (Figure 2D,E; Figure S1, Supporting Information).

### 2.2 Establishment of the Enthesis‐on‐Chip Culture

In standard tissue culture‐treated polystyrene plates (TCPs), we first assessed the multipotency potential of the hMSC donor used in the study (Figure S2, Supporting Information and preceding text in Supporting Information). Also in TCPs, we then tested combinations of selective differentiation factors (Figures S3 and S4, Supporting Information and preceding text in Supporting Information).^[^ ^30^, ^31^, ^32^, ^33^, ^34^, ^35^, ^36^, ^37^, ^38^, ^39^, ^40^, ^41^, ^42^, ^43^, ^44^ ^]^

**Figure 3:**
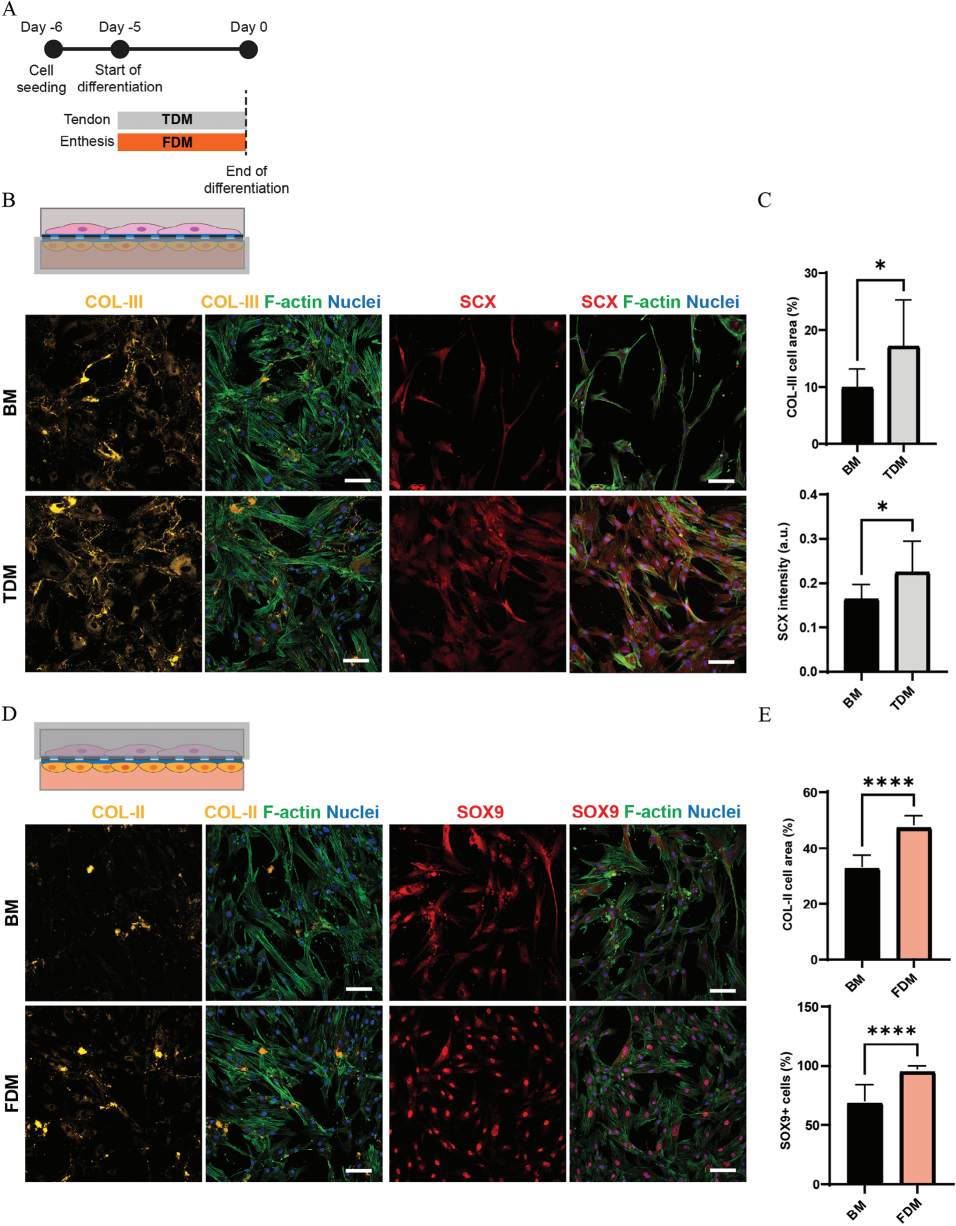
The enthesis‐on‐chip device allows simultaneous tenogenic and fibrochondrogenic differentiation. A) Schematic overview describing the timeline of the experiments. B) Confocal fluorescence images of hMSCs cultured in BM or TDM in the top compartment of the enthesis‐on‐chip device. The cells were immunostained for COL‐III (yellow) and SCX (red), and stained with phalloidin for F‐actin (green) and 4’,6‐diamidino‐2‐phenylindole (DAPI) for nuclei (blue). Scale bars represent 100 µm and apply to all images. C) Quantification of COL‐III production measured as the percentage of covered cell area and quantification of SCX mean intensity. Bars represent mean values and error bars standard deviations. Significance was determined by a two‐tailed unpaired Student's t‐test. *p < 0.05. N = 3. D) Confocal fluorescence images of hMSCs cultured in BM or FDM in the bottom compartment of the device. The cells were immunostained for COL‐II (yellow) and SOX9 (red), and stained with phalloidin for F‐actin (green) and DAPI for nuclei (blue). Scale bars represent 100 µm and apply to all images. E) Quantification of COL‐II production measured as the percentage of covered cell area and quantification of the percentage of cell nuclei stained positively for SOX9. Bars represent mean values and error bars standard deviations. Significance was determined by a two‐tailed unpaired Student's t‐test. ****p < 0.0001. N = 3.

Taken together, the corresponding results suggested a likely selective differentiation of hMSCs toward the tenogenic and fibrochondrogenic phenotype when instead of in TCPs now in the enthesis‐on‐chip device exposed to “tendon differentiation medium” (TDM) or “fibrocartilage differentiation medium” (FDM), respectively. For this, after seeding hMSCs in the chip, we simultaneously perfused the top compartment with TDM and the bottom compartment with FDM for 5 days (**Figure** **3**A). Expression of collagen (COL‐)III and scleraxis (SCX) in the top and COL‐II and the (chondrogenic) SRY‐box transcription factor 9 (SOX9) in the bottom compartment of the chip was analyzed by immunocytochemical staining. For the top compartment, quantifications revealed a significant increase of COL‐III deposition and a significant increase of SCX mean intensity in cells cultured in TDM relative to those cultured in BM (Figure 3B,C). In the bottom compartment, we measured significantly enhanced COL‐II deposition and a significantly higher number of cell nuclei positive for SOX9 in cells exposed to FDM relative to BM (Figure 3D,E). The above results suggested that the perfusion of the enthesis‐on‐chip device with TDM and FDM supports hMSCs selective differentiation toward the tenogenic and fibrochondrogenic phenotype, respectively.

### 2.3 Short‐Term Exposure to IL‐17, IL‐23 and TNF‐*α* Triggers an Acute Inflammatory Response in the Enthesis‐on‐Chip Model

During inflammation, the enthesis is exposed to waves of cytokines, which can alter cell behavior and tissue organization.^[^ ^3^ ^]^ Enthesitis often originates from microdamages in the fibrocartilage region of the enthesis, which lead to the formation of blood vessels that act as entry points for inflammatory factors. This process is sufficient to establish an inflammatory response, which can further propagate to the body of the tendon.^[^ ^45^ ^]^

**Figure 4:**
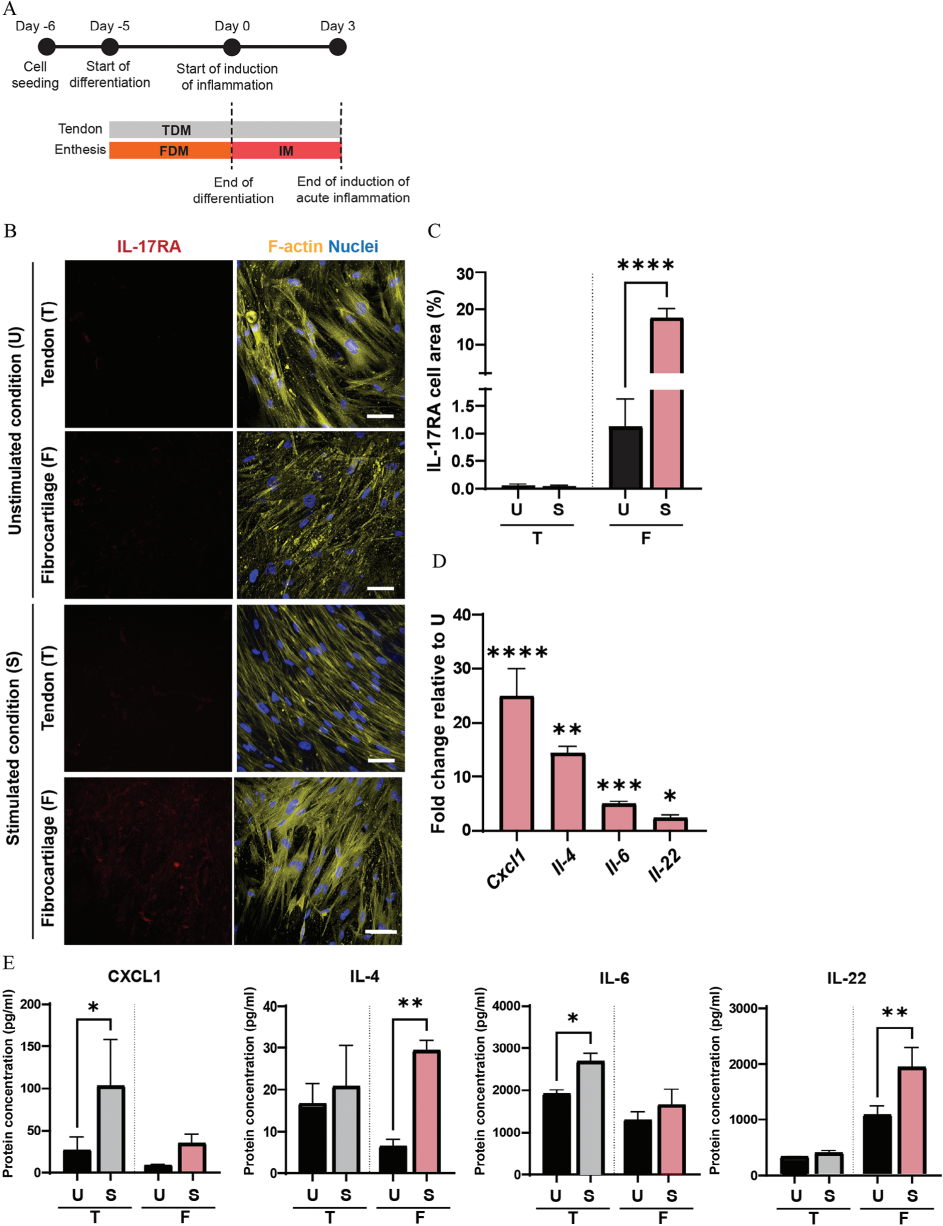
Exposure to IL‐17, IL‐23 and TNF‐*α* triggers an acute inflammatory response in the enthesis‐on‐chip device. A) Schematic overview describing the timeline of the experiments. B) Confocal fluorescence images showing the expression of IL‐17RA in the tendon (T) and fibrocartilage compartment (F) of the enthesis‐on‐chip device, after 3 days of culture in the unstimulated (U) and the inflammation‐stimulated condition (S). Cells were stained for IL‐17RA (red), cytoskeletal F‐actin filaments (yellow) and cell nuclei (blue). Scale bars represent 100 µm and apply to all images. C) Quantification of the percentage of cell area positive for IL‐17RA. Bars represent mean values and error bars standard deviations. Significance was determined by a two‐tailed unpaired Student's t‐test. ****p < 0.0001. N = 3. D) Bar graphs showing the expression of inflammatory genes in the fibrocartilage compartment of the enthesis‐on‐chip device. Bars represent mean values and error bars standard deviations. Significance was determined by a two‐tailed unpaired Student's t‐test. *p < 0.05, **p < 0.01, ***p < 0.001 and ****p < 0.0001. N = 3. E) Bar graphs showing multiplex analysis of the released levels of inflammatory proteins measured in the tendon (T) and fibrocartilage (F) compartment of the enthesis‐on‐chip device after 3 days of culture in the unstimulated (U) and the inflammation‐stimulated condition (S). Bars represent mean values and error bars standard deviations. Significance was determined by a two‐tailed unpaired Student's t‐test. *p < 0.05 and **p < 0.01. N = 3.

We aimed at reproducing enthesitis in our chip, by challenging the fibrocartilage layer in the bottom compartment with an inflammatory medium (IM), composed of FDM additionally containing IL‐17, IL‐23 and TNF‐*α*, after the induction of cell differentiation. To recapitulate acute enthesitis, we delivered IM under flow for 3 days (**Figure** **4**A).

Considering that IL‐17 has a key role in the pathogenesis of enthesitis,^[^ ^46^ ^]^ cells from both compartments were immunostained for the IL‐17 receptor IL‐17RA (Figure 4B), an inflammatory marker expressed in nearly every cell type of the body and activated upon inflammatory stimulation. Then, the corresponding fluorescence images were quantified (Figure 4C).^[^ ^47^ ^]^ After 3 days of exposure to IM, the IL‐17RA staining showed a 16.5% increase in the fibrocartilage compartment when compared to the unstimulated condition. However, IL‐17RA staining was not detectable in the tendon compartment upon exposure to IM.

To confirm the induction of acute enthesitis, cells in the fibrocartilage compartment of the chip were harvested and the mRNA expression levels of the inflammatory CXC motif chemokine ligand 1 (*Cxcl1*) and interleukins *Il‐4*, *Il‐6* and *Il‐22* were quantified by real‐time quantitative PCR (RT‐qPCR) (Figure 4D). In cells exposed to IM in the fibrocartilage compartment, we observed a significant upregulation of the expression levels of *Cxcl1* (25.0‐fold), *Il‐4* (14.5‐fold), *Il‐6* (4.97‐fold) and *Il‐22* (2.47‐fold) relative to FDM.

Moreover, due to the potential of the platform to recreate two independent microenvironments, medium from the top and bottom compartment of the chip was collected and analyzed for the expression of the released abovementioned pro‐inflammatory mediators via a multiplex assay (Figure 4E). At the protein level, with IM in the fibrocartilage compartment, we observed a significantly increased release of IL‐4 and IL‐22 in this compartment relative to the unstimulated culture. In addition, again with IM in the fibrocartilage compartment and relative to the unstimulated reference, enhanced levels of CXCL1 and IL‐6 were measured in the tendon compartment.

The chosen inflammatory markers have an established role in the progression of enthesitis. *Cxcl1*/CXCL1 levels increase in fibroblasts stimulated with IL‐17A both at the mRNA and protein level,^[^ ^47^ ^]^ while in a mouse enthesitis model IL‐23 induces *Cxcl1* by day 5, which supports bone remodeling at later time points.^[^ ^9^ ^]^ IL‐4 mainly promotes acute inflammatory processes and contributes to the alteration of the original tendon ECM architecture.^[^ ^48^ ^]^ IL‐6, which is released as a consequence of IL‐17 stimulation, promotes inflammatory mediators and bone‐degrading cytokines.^[^ ^49^ ^]^ IL‐22 is mainly involved in chronic enthesitis, where it supports new bone formation, enhancing proliferation, migration and the osteoblast differentiation potential of MSCs.^[^ ^13c^ ^]^

When looking at the above‐discussed cell response to the induction of acute enthesitis, we measured an upregulation of all genes tested in the fibrocartilage compartment of the chip, which was only partially observed at the protein level. This discrepancy between mRNA and protein expression profiles might originate from post‐transcriptional mechanisms regulating the expression^[^ ^50^ ^]^ of CXCL1 and IL‐6, which might delay the protein production process in fibrochondrocytes at the time point analyzed. Moreover, the multiplex assay showed increased release of CXCL1 and IL‐6 in the tendon compartment of the device, while IL‐4 and IL‐22 were enhanced in the fibrocartilage compartment. This evidence highlights cell type‐specific differences in the response to inflammation. Indeed, CXCL1 is one of the main upregulated pro‐inflammatory markers early expressed in diseased tenocytes induced by IL‐17 or TNF‐*α*,^[^ ^51^ ^]^ while IL‐6 is upregulated in biopsies of diseased tendon.^[^ ^52^ ^]^ On the other hand, IL‐4, a mediator of acute inflammatory processes, and IL‐22, the expression of which is mainly induced by IL‐23, are upregulated in the fibrocartilage compartment, directly exposed to the inflammatory cocktail. The above results provide evidence of communication between the top and bottom compartments of the enthesis on chip already at an early time point in the inflammation process.

### 2.4 Prolonged Exposure to IL‐17, IL‐23 and TNF‐*α* Triggers a Chronic Inflammatory Response in the Enthesis‐on‐Chip Model

**Figure 5:**
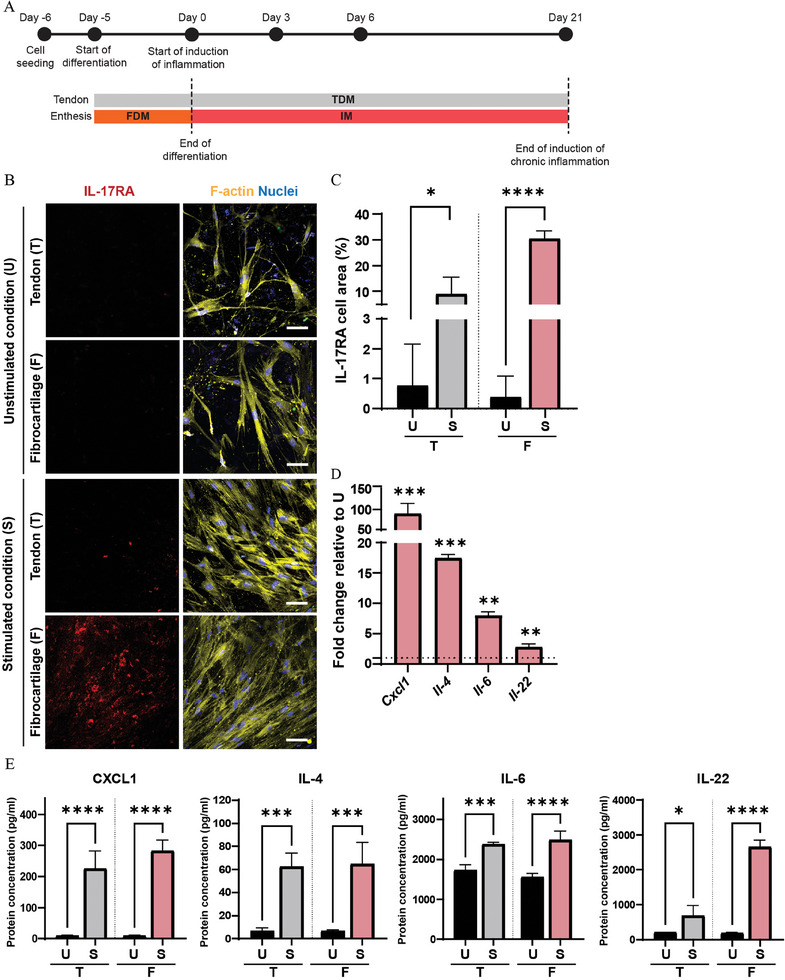
Prolonged exposure to IL‐17, IL‐23 and TNF‐*α* triggers a chronic inflammatory response in the enthesis‐on‐chip device. A) Schematic overview describing the timeline of the experiments. B) Confocal fluorescence images showing the expression of IL‐17RA in the tendon (T) and fibrocartilage compartment (F) of the enthesis‐on‐chip device after 21 days of culture in the unstimulated (U) and the inflammation‐stimulated condition (S). Cells were stained for IL‐17RA (red), cytoskeletal F‐actin filaments (yellow) and cell nuclei (blue). Scale bars represent 100 µm and apply to all images. C) Quantification of the percentage of cell area positive for IL‐17RA. Bars represent mean values and error bars standard deviations. Significance was determined by a two‐tailed unpaired Student's t‐test. *p < 0.05 and ****p < 0.0001. N = 3. D) Bar graph showing the expression of inflammatory genes in the fibrocartilage compartment of the enthesis‐on‐chip device. Bars represent mean values and error bars standard deviations. Significance was determined by a two‐tailed unpaired Student's t‐test. **p < 0.01 and ***p < 0.001. N = 3. E) Bar graphs showing multiplex analysis of the released levels of inflammatory proteins measured in the tendon (T) and fibrocartilage (F) compartment of the enthesis‐on‐chip device after 21 days of culture in the unstimulated (U) and the inflammation‐stimulated condition (S). Bars represent mean values and error bars standard deviations. Significance was determined by a two‐tailed unpaired Student's t‐test. *p < 0.05, ***p < 0.001 and ****p < 0.0001. N = 3.

To model chronic enthesitis, we perfused the fibrocartilage compartment of the chip with IM for 21 days (**Figure** **5**A). We stained cells from both compartments for IL‐17RA and observed a 30.3% increase of the cell area positive for IL‐17RA in the fibrocartilage compartment relative to the unstimulated culture (Figure 5B,C). However, we also measured a 11.4% increase of IL‐17RA in the tendon compartment under this condition.

Time course experiments also showed a significant increase in the percentage of cell area positive for IL‐17RA in both the tendon and fibrocartilage compartment at day 21 relative to day 3 (Figure S5, Supporting Information). These data suggested a restricted effect of IL‐17RA activation in the fibrocartilage compartment at day 3, which propagates to the tendon layer at day 21. The secretion of small inflammatory mediators, such as cytokines, nitric oxide, prostaglandins and lipoxins, might be involved in the propagation of an inflammatory response from the fibrocartilage compartment through the porous culture membrane to the tendon compartment.

The increased expression of IL‐17RA in both acute and chronic inflammatory conditions represents a starting point for establishing our model. Previous works showed that IL‐17 is upregulated during the early inflammatory phases and mediates the release of IL‐6 and IL‐8, key amplifiers of the inflammatory reaction.^[^ ^49^ ^]^ In chronic inflammation, IL‐17 activates the production of matrix metalloproteinases (MMPs), which lead to the degradation of different types of collagen and other matrix proteins.^[^ ^47^ ^]^ IL‐17 mediates inflammation and tissue remodeling also in conjunction with human tenocytes in both in vitro and in vivo models,^[^ ^48^ ^]^ which can justify the cell response observed at day 21 in the top compartment of the chip.

After induction of chronic inflammation in the enthesis on chip, we also measured a robust significant increase in gene expression levels of the inflammatory mediators *Cxcl1* (98.3‐fold), *Il‐4* (17.4‐fold), *Il‐6* (8.05‐fold) and *Il‐22* (2.91‐fold) in the fibroblast compartment relative to the unstimulated condition (Figure 5D). At the protein level (Figure 5E), the same inflammatory markers also displayed a significantly higher release in IM relative to the unstimulated condition in both the tendon and the fibrocartilage compartment.

The observed upregulation of all markers both at the gene and protein level indicated that the extended exposure time to the inflammatory cocktail is necessary to induce a global tissue response. In line with our observation, molecular profiling of inflamed mice enthesis tissue induced by prolonged expression of IL‐23 showed upregulation of CXCL1, IL‐6 and IL‐22.^[^ ^9^ ^]^

To check the progression of inflammation over time, we compared gene expression levels and protein concentrations at day 3 and 21 (Figure S6, Supporting Information). *Cxcl1, Il‐4* and *Il‐6* mRNA expression levels significantly increased at the later time point, while *Il‐22* gene expression levels appeared to be rather constant between the analyzed time points (Figure S6A, Supporting Information). CXCL1 and IL‐22 protein levels significantly increased at day 21 relative to day 3 in both the tendon and fibrocartilage compartment, while IL‐4 protein levels did not change between the time points analyzed in the two compartments (Figure S6B, Supporting Information). IL‐6 was significantly downregulated at day 21 relative to day 3 in the tendon compartment, while similar expression levels were measured in the fibrocartilage compartment.

Although IL‐4 is involved in acute inflammatory processes, its role in enthesitis is still not completely understood. Instead, the function of IL‐4 in RA has been the focus of intensive investigations. In such conditions, IL‐4 is upregulated in the synovial fluid of early RA and its expression is lost after prolonged inflammation. IL‐4 in RA has also an anti‐inflammatory effect, inhibiting cartilage damage and osteoclastogenesis.^[^ ^53^ ^]^ Since we measured constant IL‐4 expression levels between acute and chronic inflammation, we could hypothesize that IL‐4 may play a similar role in enthesitis, although further characterizations are necessary.

### 2.5 Prolonged Exposure to IL‐17, IL‐23 and TNF‐*α* Triggers an Osteogenic Response in the Enthesis‐on‐Chip Model

In chronic enthesitis, increased activation of the IL‐23‐IL‐17 pathway is often associated with mineral deposition, resulting in new bone formation in the fibrocartilage region of the enthesis.^[^ ^13^, ^49^ ^]^ This, in turn, disrupts the original tissue organization.

**Figure 6:**
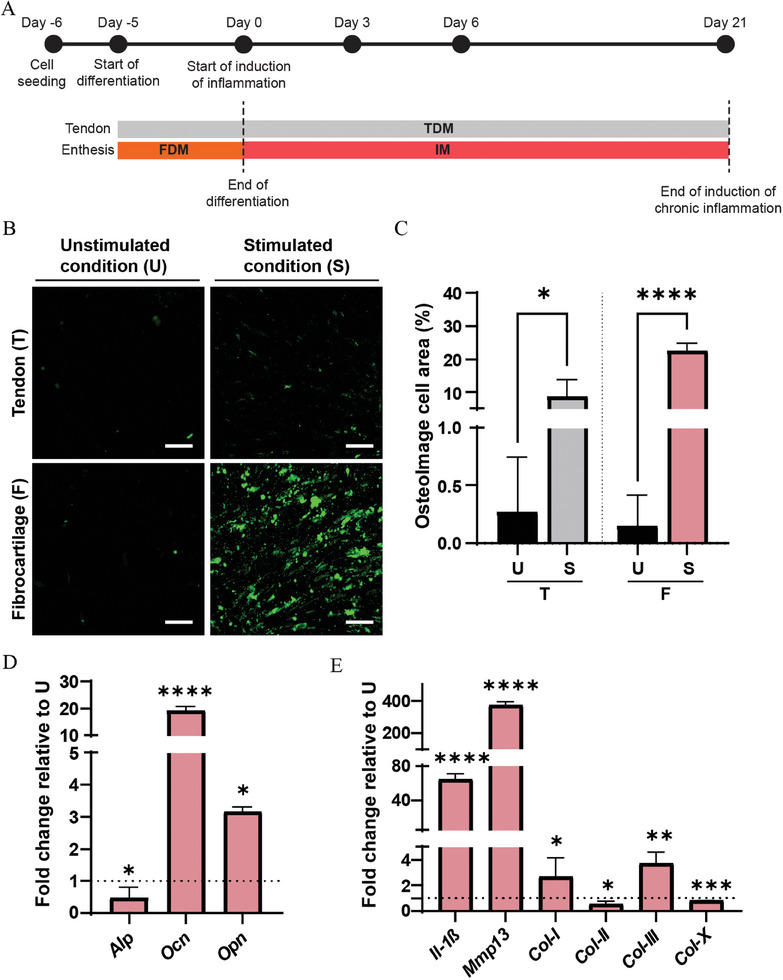
Prolonged exposure to IL‐17, IL‐23 and TNF‐*α* triggers a pro‐osteogenic response and ECM remodeling. A) Schematic overview describing the timeline of the experiments. B) Confocal fluorescence images showing the HA deposition in the tendon (T) and fibrocartilage compartment (F) of the enthesis‐on‐chip device after 21 days of culture in the unstimulated (U) and the inflammation‐stimulated condition (S). Cells were stained with OsteoImage (green). Scale bars represent 100 µm and apply to all images. C) Quantification of the percentage of cell area stained with OsteoImage. Bars represent mean values and error bars standard deviations. Significance was determined by a two‐tailed unpaired Student's t‐test. *p < 0.05 and ****p < 0.0001. N = 3. D,E) Bar graphs showing the expression of osteogenic (D) and ECM‐related genes (E) in the fibrocartilage compartment of the enthesis‐on‐chip device after 21 days of culture. Bars represent mean values and error bars standard deviations. Significance was determined by a two‐tailed unpaired Student's t‐test. *p < 0.05, **p < 0.01, ***p < 0.001 and ****p < 0.0001. N = 3.

To assess whether our enthesitis‐on‐chip model was able to reproduce this phenotype, we quantified the amount of HA deposition through OsteoImage staining after 21 days of treatment with IM (**Figure** **6**A–C). We observed a 22.6‐fold increase in HA deposition in the fibrocartilage chamber relative to FDM. Interestingly, under the same condition, we also observed a robust 9.69‐fold increase on the tendon side, suggesting that the prolonged exposure to the inflammatory cytokines has the potential to trigger osteogenic differentiation of both cell types.

To investigate whether chronic inflammation affected also cell commitment toward the osteogenic phenotype, we analyzed in the fibrocartilage compartment the mRNA expression level of alkaline phosphatase (“*Alp*”; encoding gene: alkaline phosphatase, liver/bone/kidney, *ALPL*), osteocalcin (“*Ocn*”; encoding gene: bone gamma‐carboxyglutamate protein, *BGLAP*) and osteopontin (“*Opn*”; encoding gene: secreted phosphoprotein 1, *SPP1*) (Figure 6D). *Alp* is a key mediator of mineral deposition,^[^ ^54^ ^]^ *Ocn* is expressed in tissue undergoing abnormal ossification^[^ ^55^ ^]^ and *Opn* is involved in tissue remodeling.^[^ ^56^ ^]^ Following the induction of chronic inflammation by exposure to IM, compared to FDM exposure, the *Alp* gene expression level was downregulated. This is likely because *Alp*/ALP typically appears as an early marker of osteogenic differentiation, indicating initial mineralization activity. In contrast, the expression of *Ocn* and *Opn*, which are associated with mature osteoblasts and bone matrix formation, showed a significant increase after chronic inflammation was induced. This *Ocn* and *Opn* upregulation supports the evidence of HA deposition.

Interestingly, the mRNA expression level of *Alp*, *Ocn* and *Opn* was significantly upregulated at day 21 compared to day 3 (Figure S7A,B, Supporting Information), indicating a potentially increased osteogenic commitment in the fibrocartilage region over time. Supporting this hypothesis, the enhanced *Alp* expression could be correlated with increased *Il‐22*/IL‐22 gene and protein expression. In vitro studies demonstrated that *Alp* gene expression is induced by IL‐22, which can then contribute to mineral deposition.^[^ ^57^ ^]^ In this process, *Ocn* might play a key role since it has been previously identified in enthesis samples from patients with SpA as well as in tendon undergoing abnormal ossification, although the molecular mechanisms involved in the process are still not clear.^[^ ^58^ ^]^ In addition, *Opn* upregulation could be connected with changes in matrix composition, as demonstrated in patients affected by ankylosing spondylitis.^[^ ^59^ ^]^

The above results show that prolonged treatment with the inflammatory cocktail can recapitulate key aspects observed in entheseal inflammation. In vivo, proliferation initiates the tissue response that results in new bone formation. Against this background, our platform could be beneficial to study the link between inflammation and bone formation, which remains still unclear.

### 2.6 Prolonged Exposure to IL‐17, IL‐23 and TNF‐*α* Affects ECM Composition in the Enthesis‐on‐Chip Model

Chronic inflammation is often associated with ECM destruction, which is mediated by IL‐17.^[^ ^47^ ^]^ In chondrocytes from murine knee joints, IL‐17 inhibits matrix production, leading to cartilage damage.^[^ ^60^ ^]^ IL‐17 also activates the synthesis and function of MMPs and, in combination with TNF‐*α*, leads to irreversible cartilage damage in murine models.^[^ ^9^ ^]^ Therefore, we measured the mRNA expression levels of the interleukin *Il‐1β* and the matrix‐related genes *Mmp13*, *Col‐I*, *Col‐II*, *Col‐III* and *Col‐X* in the fibrocartilage compartment of the enthesis on chip after 21 days of exposure to IM (Figure 6E).

In the fibrocartilage compartment stimulated with IM, we observed a 64.4‐fold upregulation of *Il‐1β* relative to the unstimulated condition. *Il‐1β* is a key linker between inflammation and ECM remodeling. In human spinal entheses and human tendon cell cultures, *Il‐1β* stimulates the production of other pro‐inflammatory cytokines^[^ ^61^ ^]^ and downregulates the expression of *Col I* mRNA,^[^ ^62^ ^]^ respectively. Similarly, in chondral tissue, *Il‐1β* induces matrix degeneration increasing *Mmp* expression.^[^ ^24a^ ^]^ We analyzed the expression levels of *Mmp13*, which is involved in the degradation of fibrocartilage matrix.^[^ ^63^ ^]^ *Mmp13* levels follow a similar trend as *Il‐1β*, showing a robust 391‐fold increase compared to cultures in the unstimulated condition for 21 days.

Next, we analyzed the mRNA expression levels of *Col‐I*, *Col‐II*, *Col‐III* and *Col‐X* upon IM exposure. *Col‐II* and *Col‐X*, whose corresponding proteins are main components of the ECM in healthy fibrocartilage tissue,^[^ ^64^ ^]^ were significantly downregulated compared to the unstimulated reference. However, *Col‐I* and *Col‐III*, the corresponding proteins of which are prominent in the ECM of the osteoarthritic joint,^[^ ^65^ ^]^ showed an opposite trend. The mRNA expression levels showed a statistically significant 2.69‐ and 3.80‐fold increase, respectively, compared to the unstimulated culture.

We also checked how the progression of inflammation affects ECM composition by comparing mRNA expression levels between acute and chronic inflammation (Figure S8A,B, Supporting Information). Interestingly, we observed that *Il‐1β* expression showed a 17.4‐fold increase at day 21 relative to day 3. However, *Mmp13* expression levels were downregulated by 3.32‐fold at day 21. Similarly, Lin et al. observed reduced *Mmp13* gene expression but enhanced production of the related protein in inflamed osteochondral microtissues.^[^ ^24a^ ^]^ mRNA expression levels do not directly correlate with the amount of functional protein present. Therefore, quantification of the MMP13 protein level in the culture medium could give a more accurate indication of the presence of MMP in the enthesis‐on‐chip culture.

Regarding the matrix components, we observed similar *Col‐I* and *Col‐II* gene expression levels between the two time points analyzed. *Col‐X* is significantly downregulated by 14.2‐fold at day 21 compared to day 3, while *Col‐III* is upregulated by 3.70‐fold in the chronic inflammatory condition, compared to the acute one.

This suggested that chronic inflammation in the fibrocartilage compartment of the enthesis on chip is responsible for alterations in matrix composition, thus supporting the physiological relevance of our human cell‐based enthesitis‐on‐chip model. However, it would be interesting to further assess whether the observed changes in ECM composition are also associated with the deposition of additional ECM components, such as glycosaminoglycans, or with changes in collagen fiber arrangement and diameter. The latter, in turn, may be associated with poor structural integrity. In this context, the inclusion of macrophages in the culture would give the possibility to study the matrix remodeling process during chronic enthesitis. Upon stimulation, macrophages secrete different MMPs that degrade several types of collagen and other ECM molecules,^[^ ^3^ ^]^ representing a tool to investigate enthesis matrix degradation.

Altogether, the above results suggested that our enthesis‐on‐chip model can replicate some of the key features observed in enthesis inflammation in vivo. This includes increased mineral deposition in the fibrocartilage layer and remodeling of the ECM.

### 2.7 Therapeutic Effect of CXB Administration after the End of the Induction of Acute Enthesitis in the Enthesis‐on‐Chip Device

To test the feasibility of the enthesitis‐on‐chip for drug screening applications, we introduced in the system CXB, a NSAID working as selective inhibitor of TNF‐*α*‐induced transcriptional activity.^[^ ^66^ ^]^ CXB was administered at a concentration of 10 µm, on the basis of a previous study.^[^ ^24b^ ^]^ After induction of acute or chronic inflammation, the fibrocartilage compartment of the chip was continuously perfused with CXB for 3 days. In this conjunction, we examined the cell response in the following conditions: 1) the presence of an inflammatory stimulus and in the absence of a therapeutic one (IM; “+ IM – CXB”), 2) the absence of both an inflammatory stimulus and a therapeutic one (FDM; “– IM – CXB”), 3) the absence of an inflammatory stimulus and in the presence of a therapeutic one (FDM plus CXB; “– IM + CXB”) and 4) the presence of both an inflammatory stimulus and a therapeutic one (IM plus CXB; “+ IM + CXB”).

**Figure 7:**
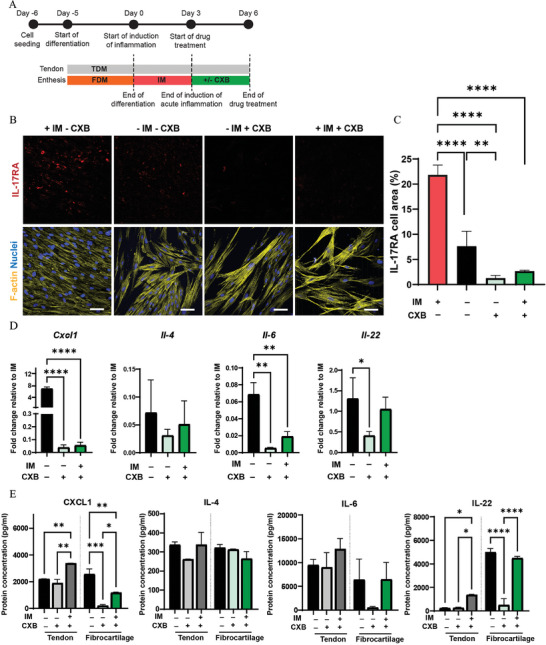
Pharmaceutical treatment with CXB decreases acute inflammation. A) Schematic overview describing the timeline of the experiment. B) Confocal fluorescence images of fibrocartilage cells after 3 days of culture in IM followed by 3 days of culture with IM and without CXB (+ IM – CXB), without IM and CXB (– IM – CXB), without IM and with CXB (– IM + CXB) and with IM and CXB (+ IM + CXB). The cells were stained for IL‐17RA (red), cytoskeletal F‐actin filaments (yellow) and cell nuclei (blue). Scale bars represent 100 µm and apply to all images. C) Quantification of the percentage of cell area positive for IL‐17RA. Bars represent mean values and error bars standard deviations. Significance was determined by one‐way ANOVA followed by Tukey's post‐hoc test. **p < 0.01 and ****p < 0.0001. N = 3. D) Bar graphs showing the expression of inflammatory genes in the fibrocartilage compartment. Bars represent mean values and error bars standard deviations. Significance was determined by a two‐tailed unpaired Student's t‐test. *p < 0.05, **p < 0.01 and ****p < 0.0001. N = 3. E) Bar graphs showing the multiplex analysis of the released levels of inflammatory proteins measured in the tendon and the fibrocartilage compartment of the enthesis‐on‐chip device. Bars represent mean values and error bars standard deviations. Significance was determined by one‐way ANOVA followed by Tukey's post‐hoc test. *p < 0.05, **p < 0.01, ***p < 0.001 and ****p < 0.0001. N = 3.

Starting with the treatment of the acute inflammation at the end of its induction (**Figure** **7**A), we first determined whether CXB affected the inflammatory response through immunocytochemistry staining for IL‐17RA (Figure 7B,C). We measured a significant 68.2% decrease in IL‐17RA expression for – IM – CXB compared to + IM – CXB, indicating that removing the inflammatory stimulus can effectively attenuate the inflammatory state. Additionally, we observed a reduction in the percentage of cell area positive for IL‐17RA when the culture medium was supplemented with CXB. Specifically, there was a significant 90.9% decrease in IL‐17RA expression for – IM + CXB and a 84.1% decrease for + IM + CXB, relative to + IM – CXB. Compared to – IM – CXB, we measured a significant 71.4% reduction in IL‐17RA cell area for – IM + CXB, while there was no statistically significant difference for + IM + CXB. The reduced expression of IL‐17RA measured in cultures supplemented with CXB (– IM + CXB and + IM + CXB) suggested that the drug can efficiently reduce the inflammatory effect of IL‐17 already at the early time point.

Next, we checked by RT‐qPCR whether the expression levels of other inflammatory markers were affected by the administration of the anti‐inflammatory drug (Figure 7D). After 3 days of CXB treatment, *Cxcl1* showed lower expression levels, with a 179‐fold decrease for – IM + CXB and a 127‐fold decrease for + IM + CXB compared to – IM – CXB. Similarly, *Il‐6* expression levels were 11.8‐fold and 3.42‐fold reduced for – IM + CXB and + IM + CXB, respectively, relative to – IM – CXB. Also *Il‐22* was downregulated for – IM + CXB, reaching a 3.18‐fold decrease relative to – IM – CXB. The *Il‐22* expression level was also reduced for + IM + CXB compared to – IM – CXB, although we did not measure a statistically significant difference. *Il‐4* expression levels were similar between the different conditions analyzed.

At the protein level, in the fibrocartilage compartment, CXCL1 and IL‐22 followed an expression pattern similar to that of the gene level (Figure 7E). We measured lower protein concentrations for – IM + CXB relative to – IM – CXB. IL‐22 showed comparable values between – IM – CXB and + IM + CXB. CXCL1 showed a reduced concentration for + IM + CXB relative to – IM – CXB. However, in the tendon compartment, we mostly measured a higher protein concentration for + IM + CXB relative to conditions lacking IM (– IM + CXB and – IM – CXB), though partly not significantly. For IL‐22, there is a significant mitigation effect for – IM + CXB compared to – IM – CXB, but only a (non‐significant) mitigation trend for – IM + CXB compared to the – IM – CXB. Quantification of IL‐4 and IL‐6 showed not significantly different concentration values between the three conditions analyzed in both the fibrocartilage and tendon compartment.

Altogether, the administration of CXB in IM in the enthesis‐on‐chip device after induction of acute enthesitis (+ IM + CXB) showed a potential to mitigate the inflammatory response, measured as a reduced IL‐17RA activation and decreased CXCL1 and IL‐22 expression levels. Additionally, we observed that solely depriving the culture of the inflammatory cocktail (– IM – CXB) is not sufficient to attenuate the inflammatory response in the fibrocartilage compartment of the chip. Compared to this condition, the administration of CXB in FDM (– IM + CXB) seems to have the higher effect on mitigating the acute inflammatory response in the fibrocartilage compartment of the chip.

### 2.8 Therapeutic Effect of CXB Administration after the End of the Induction of Chronic Enthesitis in the Enthesis‐on‐Chip Device

**Figure 8:**
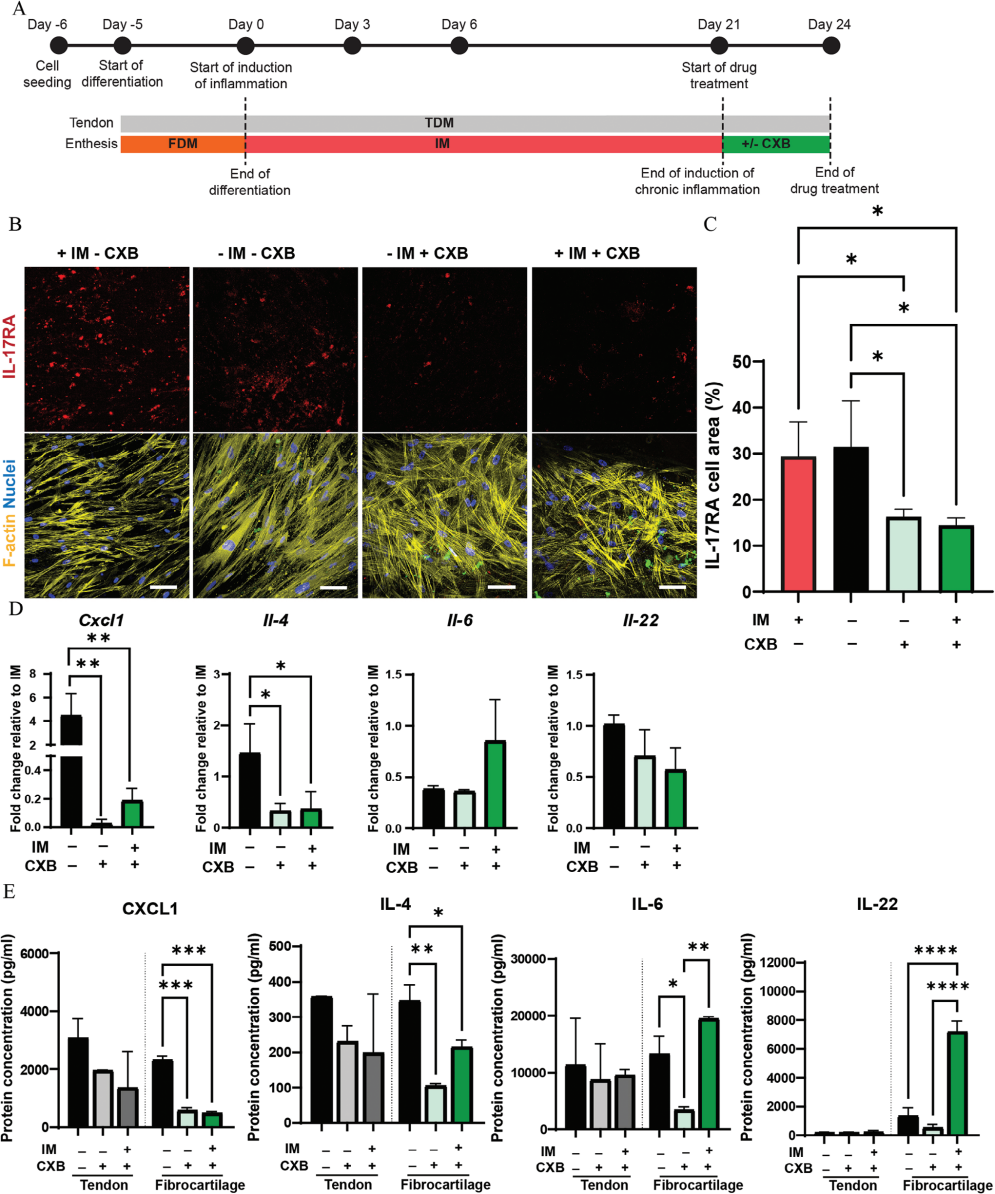
Pharmaceutical treatment with CXB reduces chronic inflammation. A) Schematic overview describing the timeline of the experiment. B) Confocal fluorescence images of fibrocartilage cells after 21 of culture in IM followed by 3 days of culture with IM and without CXB (+ IM – CXB), without IM and CXB (– IM – CXB), without IM and with CXB (– IM + CXB) and with IM and CXB (+ IM + CXB). The cells were stained for IL‐17RA (red), cytoskeletal F‐actin filaments (yellow) and cell nuclei (blue). Scale bars represent 100 µm and apply to all images. C) Quantification of the percentage of cell area positive for IL‐17RA. Bars represent mean values and error bars standard deviations. Significance was determined by one‐way ANOVA followed by Tukey's post‐hoc test. *p < 0.05. N = 3. D) Bar graphs showing the expression of inflammatory genes in the fibrocartilage compartment of the enthesis‐on‐chip device. Bars represent mean values and error bars standard deviations. Significance was determined by a two‐tailed unpaired Student's t‐test. *p < 0.05 and **p < 0.01. N = 3. E) Bar graphs showing multiplex analysis of the released levels of inflammatory proteins measured in the tendon and the fibrocartilage compartment of the enthesis on chip. Bars represent mean values and error bars standard deviations. Significance was determined by one‐way ANOVA followed by Tukey's post‐hoc test. *p < 0.05, **p < 0.01, ***p < 0.001 and ****p < 0.0001. N = 3.

After the induction of chronic inflammation (**Figure** **8**A), CXB treatment lowered the percentage of IL‐17RA signal by 50.00% for – IM + CXB and 56.67% for + IM + CXB relative to + IM – CXB (Figure 8B,C). IL‐17RA expression decreased by 53.12% for – IM + CXB and by 59.37% for + IM + CXB as compared to – IM – CXB. The gene expression levels of *Cxcl1* and *Il‐4* followed a similar pattern and were significantly suppressed in the CXB‐treated conditions (Figure 8D). Specifically, *Cxcl1* expression level showed a 224‐fold decrease for – IM + CXB and a 24.8‐fold reduction for + IM + CXB relative to – IM – CXB. *Il‐4* was 4.46‐fold downregulated for – IM + CXB and + IM + CXB relative to – IM – CXB. In contrast with the results for acute enthesitis, the *Il‐6* expression levels were not significantly different in the different conditions analyzed, and an also not significant trend of downregulation was observed for *Il‐22* in cases with CXB treatment compared to the case without.

Multiplex analysis showed significant differences in the protein content only in the fibrocartilage compartment of the chip (Figure 8E). In line with the gene expression analysis, CXCL1 and IL‐4 protein content was significantly reduced in case of CXB treatment (– IM + CXB and + IM + CXB) relative to – IM – CXB. This result is in line with a previous study where CXB significantly inhibited TNF‐*α*‐induced CXCL1 expression in the murine fibroblast cell line NIH‐3T3.^[^ ^66^ ^]^ The concentration levels of IL‐6 and IL‐22 partly showed an opposite trend. IL‐6 levels were significantly suppressed for – IM + CXB relative to – IM – CXB, but they significantly increased for + IM + CXB compared to – IM + CXB. IL‐17, IL‐23 and TNF are known to work synergistically to promote inflammation and stimulate the production of other pro‐inflammatory cytokines, including IL‐6.^[^ ^61^ ^]^ Therefore, the presence of these cytokines in the culture medium could override the anti‐inflammatory effects of CXB. Additionally, the literature indicates that IL‐6 can have anti‐inflammatory effects,^[^ ^67^ ^]^ suggesting that IL‐6 in our system may not be acting in an inflammatory role but rather in an anti‐inflammatory one.

Similarly, IL‐22 concentration significantly increased for + IM + CXB relative to the other conditions. IL‐22, rather than supporting new bone formation,^[^ ^13c^ ^]^ is also associated with tissue repair and maintenance.^[^ ^68^ ^]^ The increase of IL‐22 protein release for + IM + CXB suggests a potential tissue repair response. The interaction between CXB and the signaling pathways activated by inflammatory stimuli may result in enhanced IL‐22 production or secretion. The increased IL‐22 may represent a compensatory mechanism aimed at promoting tissue healing and maintaining tissue integrity.

Overall, after induction of chronic inflammation, CXB showed again the capacity to mitigate the inflammatory response, since it lowered IL‐17RA levels when added to the culture. In addition, we observed a downregulation of CXCL1 and IL‐4, independently of the addition of IM. The expression levels of IL‐6 and IL‐22 were mostly also reduced when CXB was added to FDM (– IM + CXB). However, the combined administration of IM and CXB (+ IM + CXB) induced higher levels of IL‐6 and IL‐22. This result is in contrast with previous reports demonstrating that CXB in combination with TNF‐*α* more effectively suppressed the pro‐inflammatory loop, measured as decreased levels of IL‐6, IL‐8, MMP1 and MMP3, in synovial fibroblasts.^[^ ^69^ ^]^ The lack of this synergetic effect in our system might be a consequence of the low concentration of the drug used or the limited duration of the treatment, which might be not sufficient to block the inflammatory response when CXB was administered together with IM (+ IM + CXB). In comparison with this condition, CXB treatment in FDM (– IM + CXB) more efficiently suppressed the inflammatory response. Lastly, we also challenged the recovery of the tissue by perfusing FDM after the induction of chronic inflammation (– IM – CXB). In such a condition, we did not observe a “rescue” of the culture, as described above for the acute inflammatory conditions.

### 2.9 CXB Treatment Affects the Osteogenic Response and ECM Composition after the End of the Induction of Chronic Enthesitis in the Enthesis‐on‐Chip Device

**Figure 9:**
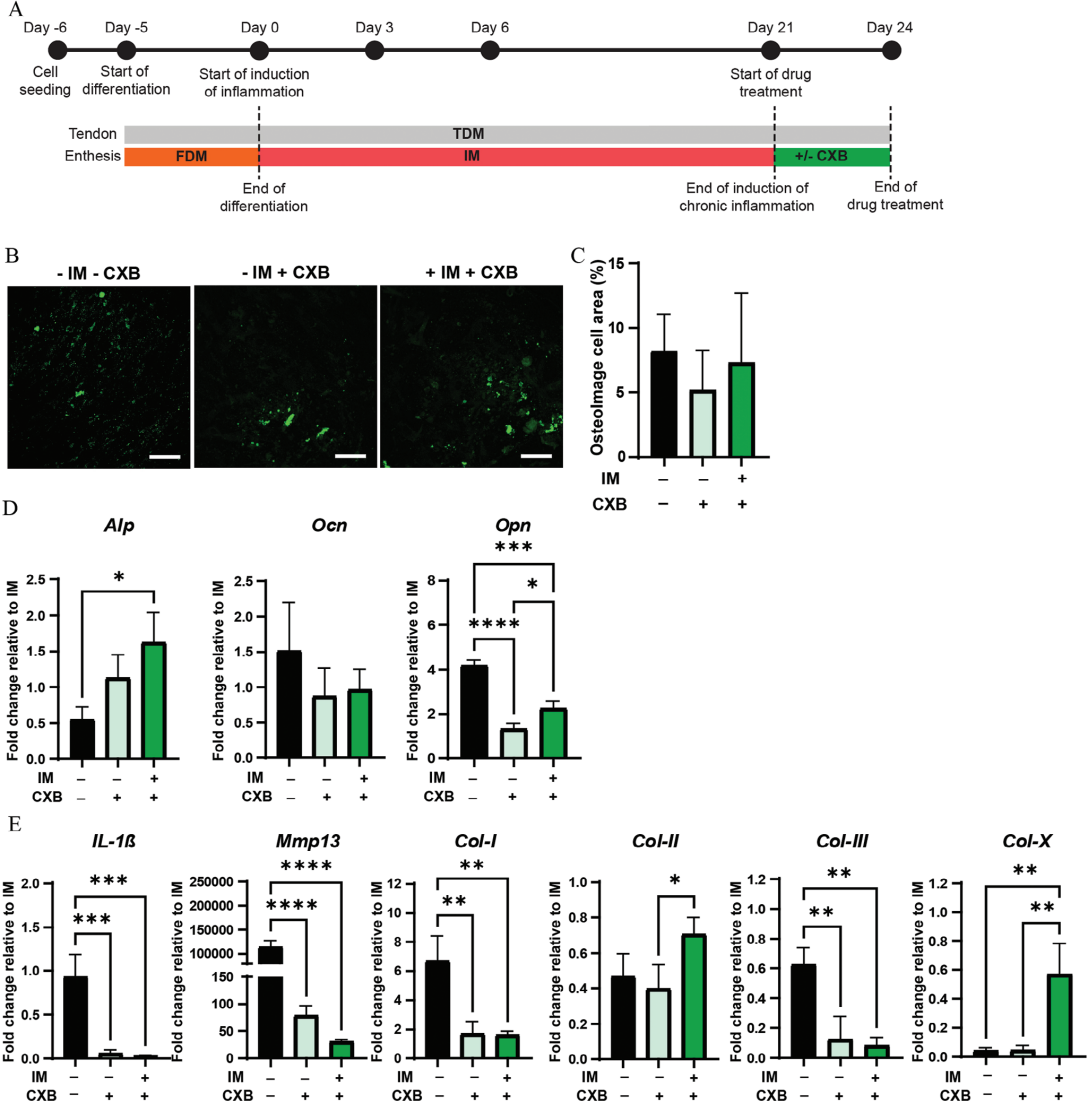
Pharmaceutical treatment with CXB affects the pro‐osteogenic response and ECM remodeling process. A) Schematic overview describing the timeline of the experiment. B) Confocal fluorescence images of fibrocartilage cells after 21 of culture in IM followed by 3 days of culture without IM and CXB (– IM – CXB), without IM and with CXB (– IM + CXB) and with IM and CXB (+ IM + CXB). Cells were stained with OsteoImage (green). Scale bars represent 100 µm and apply to all images. C) Quantification of the percentage of cell area stained with OsteoImage. Bars represent mean values and error bars standard deviations. Significance was determined by one‐way ANOVA followed by Tukey's post‐hoc test. D,E) Bar graphs showing the expression of osteogenic‐ and of ECM‐related genes in the fibrocartilage compartment of the enthesis‐on‐chip device. Bars represent mean values and error bars standard deviations. Significance was determined by a two‐tailed unpaired Student's t‐test. *p < 0.05, **p < 0.01, ***p < 0.001 and ****p < 0.0001. N = 3.

Quantification of OsteoImage staining of chronically inflamed cultures revealed similar levels of HA deposition for – IM – CXB, – IM + CXB and + IM + CXB (**Figure** **9**A–C). This suggests that the addition of CXB in the culture does not affect HA deposition at the time point analyzed. Next, we analyzed the effect of CXB on the gene expression levels of osteogenic markers (Figure 9D). *Alp* expression levels were 2.98‐fold reduced for – IM – CXB relative to + IM + CXB, while similar expression levels were observed between these two conditions and – IM + CXB. This result is in contrast with a previous study showing that CXB treatment significantly reduced *Alp* expression in the osteoblast‐like cell line MC3T3‐E1.^[^ ^70^ ^]^ This discrepancy could be due to the five times lower concentration of the drug used in our enthesitis‐on‐chip. *Ocn* expression levels were reduced for – IM + CXB and + IM + CXB compared to – IM – CXB, although no statistical differences were measured. On the contrary, *Opn* expression levels showed statistical differences in the conditions analyzed; we measured a 4.12‐fold reduced level for – IM + CXB and a 1.90‐fold decrease for + IM + CXB relative to – IM – CXB. In addition, we measured a 1.71‐fold increase for + IM + CXB relative to – IM + CXB.

A previous study demonstrated an osteoprotective effect of CXB in an osteochondral tissue chip‐based OA model, measured as a “rescue” of *Ocn* expression in the bone component of the model and compared to the non‐CXB‐treated OA model.^[^ ^24b^ ^]^ Our results on chronic enthesitis only partially replicated this. CXB exposure of the culture reduced the expression levels of *Opn* and *Ocn* statistically significantly and non‐significantly, respectively. To further promote the osteoprotective effect of CXB, it might be beneficial to increase the concentration of the drug with which the fibrocartilage compartment is perfused or the duration of the treatment.

Interestingly, in chronic enthesitis in vivo, NSAIDs often do not adequately control the disease and additional drugs are required for the treatment.^[^ ^3^ ^]^ For instance, Apremilast, a disease‐modifying anti‐rheumatic drug (DMARD), was shown to be more efficient in the treatment of chronic enthesitis since it inhibits simultaneously the production of several cytokines involved in entheseal inflammation.^[^ ^71^ ^]^ Therefore, different drugs should be tested in the enthesitis on chip for the prediction of their therapeutic efficacy.

Next, to explore the effect of CXB on ECM degeneration, we examined the expression levels of ECM‐related markers (Figure 9E). *Il‐1β*, *Mmp13*, *Col‐I* and *Col‐III* showed the same pattern and were significantly suppressed when the culture was supplemented with CXB, relative to – IM – CXB. *Col‐II* and *Col‐X* showed enhanced expression levels when culturing simultaneously in IM and CXB. *Col‐II* expression levels increased by 1.80‐fold for + IM + CXB relative to – IM + CXB. *Col‐X* reached a 13.1‐fold increase for + IM + CXB compared to – IM + CXB and a 14.2‐fold increase relative to – IM – CXB.

Previous studies on OA reported a chondroprotective effect of CXB, reducing the expression of *Il‐1β* and stimulating the synthesis of proteoglycans.^[^ ^24b^ ^]^ Similarly, we observed a significant decrease in *Il‐1β* when CXB was administered to the system. In addition, CXB suppressed the expression of *Mmp13*, which is responsible for ECM degradation in fibrocartilaginous tissue, which is also suggesting a protective effect of CXB. Besides, CXB downregulated the expression of *Col‐I* and *Col‐III*, whose corresponding proteins, as mentioned above, are prominent in osteoarthritic joints, while the drug partially increased the expression of *Col‐II* and *Col‐X*.

While the above results suggest that the enthesis‐on‐chip model is a relevant tool to recapitulate key aspects of acute and chronic enthesitis, there are some obvious limitations. First, the simplified system is lacks the bone component of the interface. Second, in vivo, different mechanical forces, such as from compression and stretch, act on the enthesis and can influence its homeostasis in both healthy and diseased conditions. In particular, the bone‐entheseal junction is subject to mechanical stress that can activate osteitis as an inflammatory reaction, which supports the formation of enthesitis, resulting in mesenchymal proliferation and osteoblast differentiation.^[^ ^3^ ^]^ In the current model, the application of mechanical stress would be only feasible by exerting higher shear stress through the application of increased flow rates, which could be also periodically modulated. Therefore, a further improvement of our current enthesis‐on‐chip model could include the development of a three‐layer microfluidic device that is not only able to host the tendon and fibrocartilage region of the enthesis but also the bone side. Applying realistic stress to the model tissue using mechanical microactuators^[^ ^72^ ^]^ could recapitulate the forces involved in the pathogenesis of enthesitis. Also, the inclusion of macrophages and/or lymphocytes, which in vivo are involved in the response to inflammatory conditions, could help to build a more relevant model of enthesitis.

## 3 Conclusion and Outlook

We have successfully developed an enthesis‐on‐chip model from hMSCs, which is capable of emulating some of the key features of acute and chronic enthesitis, after the treatment with IL‐17, IL‐23 and TNF‐*α*. Our findings have demonstrated the occurrence of a physiological cross‐talk between tenocytes and fibrochondrocytes, augmenting tendon inflammation and mineral deposition under chronic inflammatory conditions. Moreover, the anti‐inflammatory and anti‐degenerative effect of CXB was mainly replicated after induction of chronic inflammation in the enthesis‐on‐chip device, suggesting its potential future application as a drug screening platform. Additionally, our model might be used to investigate cell‐cell interactions and determine cell responses to externally applied biochemical stimuli. Future work should include the comparison of fold changes of mRNA expression levels concerning pro‐inflammatory markers as a consequence of the treatment with certain cytokines with corresponding human in vivo data where available, particularly in a spatially resolved form what concerns the tendon and fibrocartilage region of the (inflamed) enthesis.

## 4 Experimental Section

### Computational Fluid Dynamics Simulation

The medium flow through the culture chamber of the microfluidic enthesis on chip was modeled in COMSOL Multiphysics (version 5.4). The CFD simulation was run as a two‐dimensional (2D) laminar and steady/stationary flow problem. The boundary conditions at the sidewalls of the culture chamber were set to “no slip”. The culture media perfusing the culture chambers were approximated as a(n incompressible) Newtonian fluid that concerning its physical/inertial and rheological properties is identical with water at 37 °C. Under culture conditions, differences concerning the density of both fluids are practically non‐existent and regarding the (dynamic) viscosity differences are in the percent to low ten‐percent range, depending on the medium and its supplements.^[^ ^73^ ^]^ Also, as continuously fresh medium was pumped from a syringe through the chip into a waste reservoir (via tubing sections in between), the composition and consequently the flow characteristics of the medium in the culture chambers did not change over time. The volumetric flow rate was set to 4 µL h^−1^ and the (gauge) pressure at the transition from the culture chamber to the outlet channel was set to 0 Pa.

### Design, Fabrication and Permeability Testing of the Microfluidic Device

The enthesis‐on‐chip device consisted of a top and a bottom housing half made from PDMS, containing squared microfluidic chambers of 2800 × 2800 µm^2^, and a 10 µm‐thin porous PC cell culture membrane with a pore diameter of 0.4 µm and a pore density of 1 × 10^6^ pores cm^−^^2^ (it4ip). The PC membrane was sandwiched between the two housing halves. This configuration resulted in two stacked culture chambers, a top and a bottom one, which could be perfused independently through dedicated microfluidic in‐ and outlet channels and ports. Thereby, the chambers were separated by the membrane. The microfluidic chambers were designed in AutoCAD (Autodesk). The design of the culture chambers was the same for the top and the bottom housing halves, apart from the positions of the inlet and outlet channels, which were rotated by 90° between the top and the bottom structures.

For fabricating the PDMS housing halves, first, a casting mold was produced. This was done by photolithography in SU‐8 epoxy resist (NANO SU‐8 100; Micro Resist Technology) with a thickness of 75 µm on a silicon wafer (Si‐Mat Silicon Materials). The photoresist‐coated wafer was exposed to UV light through the photomask using an EVG620 mask aligner (EV Group). The SU‐8 structures on the wafer were developed by dipping in propylene glycol monomethyl ether acetate (PGMEA; Sigma–Aldrich), followed by rinsing with isopropanol and blow‐drying with nitrogen. Then, the PDMS base resin and curing agent (SYLGARD 184; Dow) were mixed in a 10:1 w/w ratio, briefly degassed in a vacuum desiccator to remove air bubbles, cast on the mold, degassed again and cured at 80 °C for 1 h. After that, the cured PDMS layer containing the microfluidic chambers was peeled off. Then, the housing halves were cut out and four microfluidics ports were punched into the top housing halve using a 730 µm diameter puncher (SYNEO). PDMS mortar was used to covalently bond and seal the two housing halves with the smaller membrane enclosed between them.^[^ ^74^ ^]^ Briefly, to create the PDMS mortar layer on the housing halves before assembly, uncured PDMS and hexane (VWR) were mixed at a ratio of 1:3 v/v and degassed in a vacuum desiccator. The hexane‐diluted PDMS was coated on a cleaned glass cover slip using a WS‐650Mz‐23NPPB spin processor (Laurell Technologies) for 3 s at 500 rpm and 60 s at 1500 rpm with a ramp of 500 rpm s^−1^ in between to generate a thin mortar layer. The cleaned PDMS housing halves were placed onto the spin‐coated PDMS mortar and allowed to stay in contact for 15 s. Afterwards, the excess mortar was removed by stamping the housing halves in a clean Petri dish and the PC membrane was placed onto one housing half. Then, the other housing half was placed onto and aligned to the membrane‐housing half assembly. The final assembly was kept at room temperature (RT) overnight to allow the PDMS mortar to cure completely.

To determine the permeability of the PC membrane, three independent experiments were performed using different anionic FITC‐labeled dextrans with MWs of 4, 40 and 70 kDa (Invitrogen). The FITC‐dextrans were provided in powder form and dissolved in PBS at a concentration of 100 µg mL^−1^. 100 µL of the dextran solution was then diluted in 1 mL of PBS and the bottom compartment of the microfluidic device was perfused with this final solution using a syringe pump at a constant flow rate of 4 µL h^−1^ for 10 days. At the same time, the top compartment was perfused with PBS at the same flow rate as the bottom compartment. The outlets of the microfluidic top and bottom compartments were connected to 1 mL tubes in which the solutions were collected at day 3, 5 and 10. After each of the three samplings, the fluorescent intensity of the solutions from the top and bottom compartments was measured with a CLARIOstar Plus microplate reader (BGM Labtech) with excitation and emission wavelengths of 485 ± 10 and 530 ± 10 nm, respectively. The dextran amounts could then be determined using a calibration curve for each of the three dextran types. The permeability testing was performed for each dextran type in triplicates.

### Cell Culture

hMSCs were isolated as previously described from bone marrow aspirate of a (single) donor^[^ ^75^ ^]^ who had given consent, based on a corresponding document from the Medical Ethical Committee (Medisch Ethische Toetsingscommissie; METC) of the Medisch Spectrum Twente hospital, Enschede, The Netherlands (study protocol “Functioneel weefselherstel met behulp vanuit beenmerg verkregen stamcellen”; K06‐002). After isolation, the cells were seeded at a density of around 1500 cells cm^−2^ in T‐flasks. There, the cells were expanded in BM, consisting of minimum essential medium (MEM) *α* without nucleotides and with Glutamax (Thermo Fisher Scientific), supplemented with 10% v/v fetal bovine serum (FBS; Sigma‐Aldrich) and 100 U mL^−1^ penicillin/streptomycin (Thermo Fisher Scientific). The cells were cultured in an incubator at 37 °C in a humidified atmosphere with 5% carbon dioxide (CO_2_). The medium was replaced every 2 or 3 days and the cells were passaged when reaching 80% confluence. The cells in this study were used at passage 3–5.

### Induction and Assessment of Adipogenic and Osteogenic Differentiation

hMSCs were seeded at a density of around 10000 cells cm^−^^2^ into a 6‐well plate and grown to confluence in BM prior to differentiation. To induce osteogenic differentiation, BM was replaced with osteogenic induction medium, composed of BM supplemented with 0.01 m *β*‐glycerophosphate, 0.2 mm ascorbic acid and 0.1 µm dexamethasone. To induce adipogenic differentiation, BM was substituted with adipogenic induction medium, composed of Dulbecco's modified Eagle medium (with high glucose level and no sodium pyruvate; Gibco) supplemented with 10% FBS, 40 mm indomethacin, 83 mm 3‐isobutyl‐1‐methylxanthine, 10 mg mL^−1^ insulin and 0.1 mm dexamethasone. The cultures were maintained for 21 days and the medium was refreshed every 2 days. Afterwards, the cells were washed twice with PBS, fixed with a 4% w/v solution of paraformaldehyde (Sigma–Aldrich) in PBS for 15 min at RT and washed three times with distilled water. To assess osteogenic differentiation, the mineralized ECM was stained with a 2% w/v solution of Alizarin Red S (VWR) in distilled water (pH = 4.2) for 15 min. In addition, HA deposition was assessed with the OsteoImage Mineralization Assay (Lonza) according to manufacturer's instructions. Adipogenic differentiation was evaluated by Oil Red O‐based lipid staining. For this, after fixation, the cells were incubated for 5 min with a 60% aqueous solution of isopropanol and the intracellular lipid accumulation was stained with Oil Red O diluted in water for 15 min. The fluorescent OsteoImage signal was acquired by confocal laser scanning fluorescence microscopy using a TCS SP8 STED (stimulated emission depletion) microscope (Leica Microsystems), the other images were taken with an Eclipse TS100 inverted microscope (Nikon).

### Cell Seeding and Microfluidic Culture in the Enthesis‐on‐Chip Device

Before cell culture, the microfluidic compartments were sterilized overnight with a 70% aqueous solution of ethanol and three times washed in PBS for 30 min. The porous membrane between the housing halves was then coated with a PureCol bovine type I collagen solution (Advanced BioMatrix; 3.0 mg mL^−1^; pH ≈2). Briefly, the chilled collagen solution was mixed with chilled 10X MEM (Thermo Fisher Scientific) in a 8:1 v/w ratio and adjusted to physiological pH with sterile 0.1 m sodium hydroxide. The solution was then diluted in sterile water to a final collagen concentration of 0.5 mg mL^−1^. The diluted collagen solution was first infused into the bottom compartment of the chip and allowed to jellify at 37 °C for 90 min. After three washes with PBS, hMSCs were seeded on the bottom side of the functionalized membrane at a density of around 5000 cells cm^−^^2^ by infusion of a suspension of cells in BM into the bottom compartment of the microfluidic chip. BM without cells was infused into the chip's top compartment. The cells were allowed to settle on and adhere to the culture membrane for 4 h. During this time interval, the device and with it the membrane was turned and kept upside‐down at 37 °C. To prevent the cells from detaching during dynamic culturing and provide them with a semi‐3D environment, the diluted collagen solution was again gently infused into the bottom compartment of the chip and incubated at 37 °C for 30 min. Next, filter (pipette) tips as temporary medium reservoirs and containing 10 µL of BM were press‐fitted into the ports connected to the bottom compartment of the chip. A corresponding coating, seeding and (again) coating/embedding procedure was then also carried out for the top compartment of the chip.

Then, both chip compartments were perfused with the appropriate media with a flow rate of 4 µL h^−1^ using 1 mL syringes mounted in a rack of a syringe pump (World Precision Instruments) and through tubes with an inner diameter of 500 µm and an outer diameter of 1.5 mm (Tygon, SynVivo), press‐fitted into the microfluidic ports of the chip. During cell culture, the chips were kept inside an incubator at 37 °C in a humidified atmosphere with 5% CO_2_.

### Cell Labeling

Prior to co‐culture in the enthesis‐on‐chip device, the hMSCs were labeled with colored cell trackers to visualize how the cells reside on the two sides of the PC membrane. The cells in the top compartment were treated with 0.1 µm CellTracker Red CMTPX (Thermo Fisher Scientific) and the cells in the bottom compartment with 0.1 µm CellTracker Green CMFDA (Thermo Fisher Scientific). Briefly, after cell culture in TCPs, the BM was replaced with the pre‐warmed CellTracker working solution and incubated for 30 min at 37 °C. Next, the cells were washed twice, trypsinized and seeded first in the bottom and then in the top compartment of the microfluidic device, as described above. The cells were kept in culture under perfusion for 5 days. Afterwards, the cells were washed with PBS, fixed with a 4% w/v paraformaldehyde solution at RT for 20 min and imaged using confocal fluorescence microscopy.

### Induction of Tenogenic and Fibrochondrogenic Differentiation

Tenogenic and fibrochondrogenic differentiation was first evaluated for hMSCs cultured in a 6‐well plate. Prior to cell seeding, the bottom of the culture wells was coated with the 0.5 mg mL^−1^ collagen type I solution described above and the collagen was allowed to jellify at 37 °C for 90 min. hMSCs were then seeded in BM at a density of around 5000 cells cm^−^^2^ and were allowed to settle and adhere for 4 h at 37 °C. Next, 200 µL of the collagen type I solution was gently added on the top of the cells to recreate the sandwich system described above for the on‐chip culture. After 30 min, the BM was removed and replaced with TDM or FDM. The cultures were maintained for up to 8 days. TDM consisted of MEM *α* without nucleotides and with Glutamax (Thermo Fisher Scientific), supplemented with 1% v/v sodium pyruvate (Gibco), 100 U mL^−1^ penicillin/streptomycin, 2% v/v FBS, 1% v/v Insulin‐Transferrin‐Selenium (ITS; Thermo Scientific) and 10 ng mL^−1^ TGF‐*β*2 (PeproTech). FDM consisted of Dulbecco's Modified Eagle Medium (with high glucose level and no sodium pyruvate; Thermo Fisher Scientific), supplemented with 100 U mL^−1^ penicillin/streptomycin, 2% v/v FBS, 1% ITS, 50 µg mL^−1^ L‐ascorbic acid 2‐phosphate (Sigma), 10 ng mL^−1^ TGF‐*β*2 and 20 ng mL^−1^ GDF5 (PeproTech).^[^ ^30b^ ^]^

During the microfluidic cultures, the top compartment of the chip was perfused with TDM and the bottom one with FDM using a flow rate of 4 µL h^−1^. The chips were kept in culture for 5 days inside an incubator at 37 °C in a humidified atmosphere with 5% CO_2_. The medium‐filled 1 mL syringes mounted in a rack of a syringe pump (World Precision Instruments) were changed every 2 days.

### Simulation of Acute and Chronic Enthesitis

To simulate inflammatory conditions in the enthesis on chip, IL‐17, IL‐23 and TNF‐*α* (all PeproTech) were added to the FDM at a concentration of 10 ng mL^−1^ each. Cytokine concentrations were carefully chosen, first, as a starting point, based on the literature, from the closest possible study fields since there was no study on a comparable enthesitis in vitro model. The studies report on 10 ng mL^−1^ IL‐23 in conjunction with entheseal inflammation,^[^ ^9^ ^]^ 10 ng mL^−1^ IL‐17A in conjunction with tendon inflammation,^[^ ^76^ ^]^ 10 ng mL^−1^ IL‐17A in conjunction with OA^[^ ^77^ ^]^ and 10 ng mL^−1^ TNF‐*α* in conjunction with RA and OA.^[^ ^78^ ^]^ Then, additionally, multiple concentrations as well as combinations of cytokines in the stimulation cocktail were tested prior to selecting the most optimal in terms of inducing an inflammatory state in the cells. The bottom compartment of the chip was perfused with IM at a flow rate of 4 µL h^−1^ for 3 or 21 days after cell differentiation, to induce acute and chronic enthesitis, respectively. The syringes containing IM were replaced every 2 days.

### Drug Testing After Induction of Acute and Chronic Enthesitis on Chip

Lyophilized CXB (PHR1683; Sigma–Aldrich) was dissolved and diluted in dimethyl sulfoxide (DMSO; Sigma–Aldrich) to a concentration of 1 mm. Stock solutions were stored at −20 °C and, after thawing, freshly diluted in FDM to a final concentration of 10 µm. Next, the diluted CXB solution was sterilized using a 0.22 µm syringe filter and the bottom compartment of the microfluidic chip was perfused with it at a flow rate of 4 µL h^−1^ for 3 days after induction of a simulated acute or chronic enthesitis.

### RNA Isolation and Gene Expression Analysis

Total RNA was isolated using the RNeasy Mini Kit (QIAGEN) according to manufacturer's protocol. For the microfluidic cultures, five individual chips were pooled together and used to extract mRNA from only the bottom chamber of the device. Briefly, 10 µL of lysis buffer solution was flushed 3–4 times through the culture chamber and then stored at −80 °C. Next, RNA purity and concentration was measured using a BioDrop µLITE (BioChrom). Complementary DNA (cDNA) was synthesized with the iScript cDNA synthesis kit (Bio‐Rad), starting from 200 ng of RNA for TCPs and 10 ng of RNA for on‐chip samples. Gene expression analysis was performed by RT‐qPCR using the iQ SYBR Green Supermix (Bio‐Rad) in a CFX96 Real‐Time PCR Detection Kit (Bio‐Rad) amplifying 20 ng of cDNA. Quantification of transcription levels was performed through the ΔΔCt method using glyceraldehyde 3‐phosphate dehydrogenase (GAPDH) or 18S ribosomal RNA (18S rRNA) as housekeeping gene. Results are presented as gene expression levels relative to their reference condition. The sequences of primers for each gene are listed in Table S1 (Supporting Information).

### Fluorescence Staining and Confocal Fluorescence Microscopy

The cells were fixed with a 4% paraformaldehyde solution at RT for 20 min, washed three times with PBS and permeabilized with 0.1% v/v Triton X‐100 (Acros Organics) at RT for 10 min. After three washing steps, non‐specific binding sites were blocked in CAS‐Block Histochemical Reagent (Thermo Fisher Scientific) at RT for 30 min. Primary antibodies, diluted in the CAS‐Block solution, were added to the cells and incubated overnight at 4 °C. A list of the used primary antibodies and their concentrations/dilutions is provided in Table S2 (Supporting Information). Next, the samples were washed three times with PBS and incubated with the secondary antibodies for 1 h at RT. The secondary antibodies and their dilutions are listed in Table S3 (Supporting Information). F‐actin was stained with phalloidin conjugated with Alexa Fluor 488 or Alexa Fluor 647 (Thermo Fisher Scientific; dilution 1:100) and cell nuclei were stained with DAPI (Sigma–Aldrich; dilution 1:100). For this, the cells were incubated for 30 min at RT. The cells of chips from chronic inflammation cultures were before and after CXB treatment additionally stained with OsteoImage, according to manufacturer's protocol, to visualize HA deposits.

After the staining, the chips were disassembled using a blade and the cultured membranes were mounted under cover slips with Dako Fluorescent Mounting Medium (Agilent). The samples were imaged by confocal fluorescence microscopy using a TCS SP8 STED microscope (Leica Microsystems). The images were acquired as z‐stacks with slices every 1 µm at a 25x magnification using a water immersion objective.

### Image Analysis

To evaluate cell differentiation and cell response to IM and to CXB treatment, microscopy images were analyzed using semi‐automated image analysis in CellProfiler (https://cellprofiler.org/; version 4.2.0) based on customized pipelines.^[^ ^79^ ^]^ Briefly, in each pipeline, the nucleus morphology was identified using the Otsu adaptive thresholding on the DAPI channel and the cell morphology was determined using the propagation algorithm in combination with the Otsu adaptive thresholding on the phalloidin channel. Cells touching the edges of the images were excluded from the dataset. After background correction, COL‐II, COL‐III, IL‐17RA and OsteoImage stainings were quantified as the pixel area stained by the input marker relative to the total segmented cell area. SCX intensity values of each pixel inside the segmented cell area were used to calculate the integrated SCX value. This value was then divided by the cell area in pixels. The percentage of SOX9‐positive cells was determined by counting the number of positive nuclei and dividing this number by the total number of nuclei identified in the image.

### Multiplex Assay

A protein panel (ProcartaPlex custom panel, Invitrogen) containing CXCL1, IL‐4, IL‐6 and IL‐22 was performed at day 3 and 21 according to the manufacturer's instructions in culture medium supernatant. Therefore, 50 µL of supernatant, along with standards, was added to antibody‐coupled beads, shaken for 30 min, incubated overnight at 4 °C and shaken again for 30 min. Next, biotin‐coupled detection antibodies were added to the beads and incubated for 30 min. The beads were then incubated with streptavidin‐phycoerythrin for 30 min while shaking. The beads were washed between the steps. protein concentrations were measured on a Luminex 100 system (Bio‐Rad) and data was acquired using Bio‐Rad Bio‐Plex Manager (version 6.0). Before each run, the system was calibrated. Standard curves were established for different analytes with known concentrations of proteins to extrapolate the protein concentrations from the measurements.

### Statistical Analysis

Each set of samples contained three independent replicates (for example, cell populated membranes from three different chips) per experimental condition (N = 3). The quantified data are presented as means ± standard deviations. Statistical analyses were performed using GraphPad Prism (version 8.4.2).

The intensity quantification of FITC‐dextrans with which the enthesis‐on‐chip device was perfused was analyzed using two‐way ANOVA followed by Tukey's post‐hoc test. For evaluating cell differentiation and the effect of IM in the cultures, quantitative differences in immunohistochemistry results between the experimental groups and the reference group were measured using a two‐tailed unpaired Student's t‐test. Data from CXB treatment were analyzed using one‐way ANOVA in combination with Tukey's post‐hoc test. RT‐qPCR experiments were analyzed using a two‐tailed unpaired Student's t‐test. Multiplex data were analyzed with one‐way ANOVA in combination with Tukey's post‐hoc test. Time course experiments were analyzed with a two‐tailed unpaired Student's t‐test between day 3 and day 21.

## Conflict of Interest

The authors declare no conflict of interest.

## Author Contributions

S.G., P.H. and R.T. contributed equally to this work. F.G. performed formal analysis, investigation, methodology, validation, visualization, wrote the original draft, wrote, review & edited the final manuscript. H.S.R. performed investigation, wrote, review & edited the final manuscript. M.E.‐L. performed investigation, wrote, review & edited the final manuscript. Z.T.B. performed methodology, supervision, validation, wrote, review & edited the final manuscript. C.B. performed funding acquisition, wrote, review & edited the final manuscript. M.v.G. performed methodology, supervision, wrote, review & edited the final manuscript. S.G. performed conceptualization, funding acquisition, methodology, supervision, validation, wrote, review & edited the final manuscript. P.H. performed conceptualization, funding acquisition, methodology, supervision, validation, wrote, review & edited the final manuscript. R.T. performed conceptualization, funding acquisition, methodology, supervision, validation, wrote, review & edited the final manuscript.

## Supporting information

Supporting Information

## Data Availability

The data that support the findings of this study are available from the corresponding author upon reasonable request.
