# Supplementary material for: Enthesitis on Chip – A Model for Studying Acute and Chronic Inflammation of the Enthesis and its Pharmacological Treatment
Source: Adv Healthc Mater. 2024 Aug 27;13(31):2401815. doi: 10.1002/adhm.202401815 (PMC11650547; doi:10.1002/adhm.202401815)
Supplement: Supplementary file 1 — Supporting Information [file ADHM-13-0-s001.docx]

**Supporting Information**

Enthesitis on Chip – a Model for Studying Acute and Chronic Inflammation of the Enthesis and its Pharmacological Treatment

*Francesca Giacomini, Hoon Suk Rho, Maria Eischen-Loges, Zeinab Tahmasebi Birgani, Clemens van Blitterswijk, Martijn van Griensven, Stefan Giselbrecht†, Pamela Habibović†, Roman Truckenmüller†**

†these authors equally contributed to this work


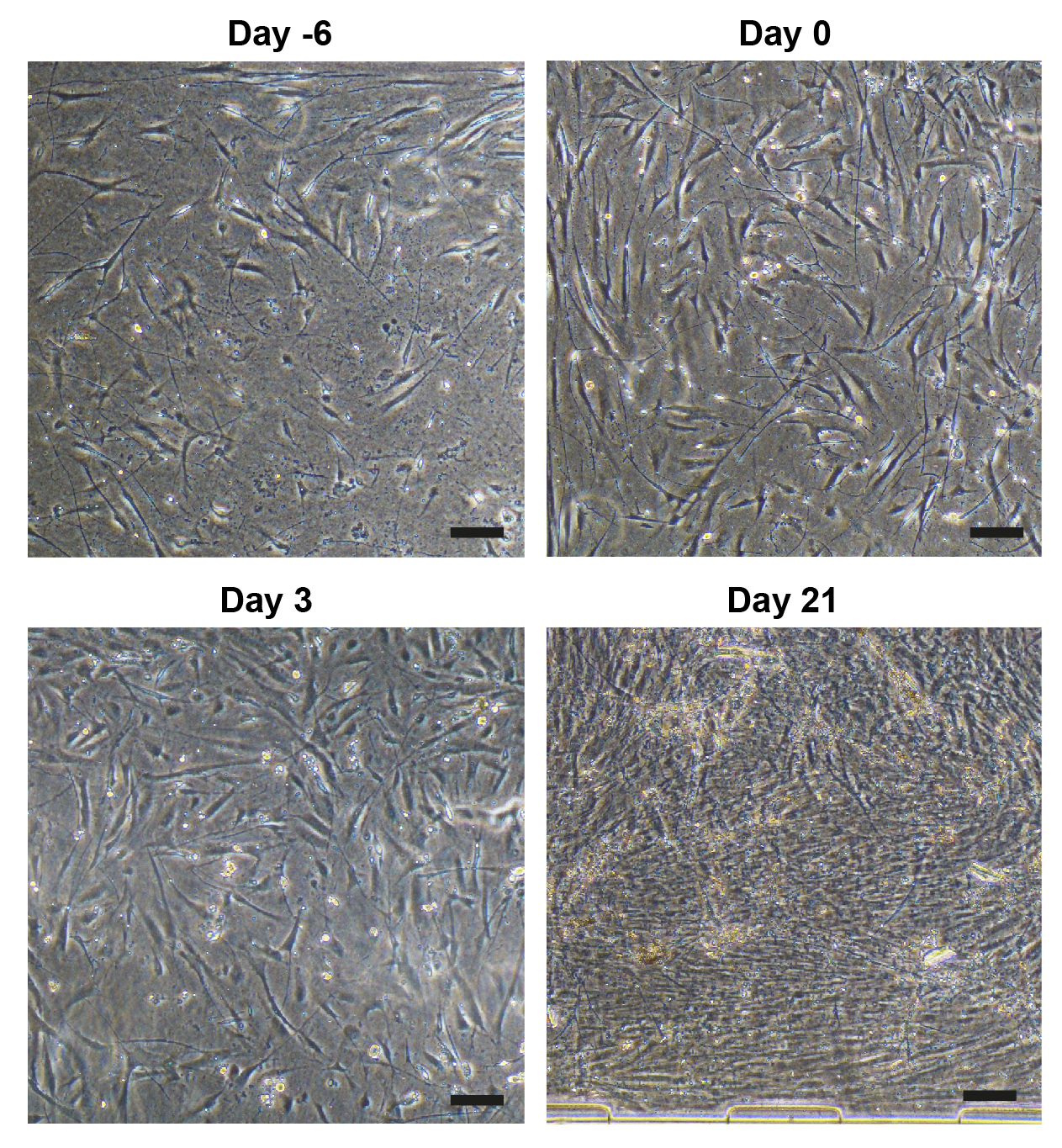


**Figure S1.** Bright-field microscopy images of human mesenchymal stromal cells (hMSCs) seeded in the enthesis on chip. Images were acquired at the day of seeding (day -6) and after 6, 9 and 27 days of culturing in basic medium (BM) under flow in a collagen sandwich system. Scale bars represent 100 µm.

We first assessed the multipotency potential of the hMSC donor used in the study (**Figure S2**). When cultured in osteogenic medium (OM) in standard tissue culture-treated polystyrene plates (TCPs) for 21 days, the cells exhibited increased hydroxyapatite (HA) deposition, observed through Alizarin Red and OsteoImage staining. Cells cultured in adipogenic medium (AM) in TCPs for 21 days began to accumulate intracellular lipid vacuoles that progressively filled the cytoplasm and were positive for Oil Red O staining, confirming their adipogenic phenotype.


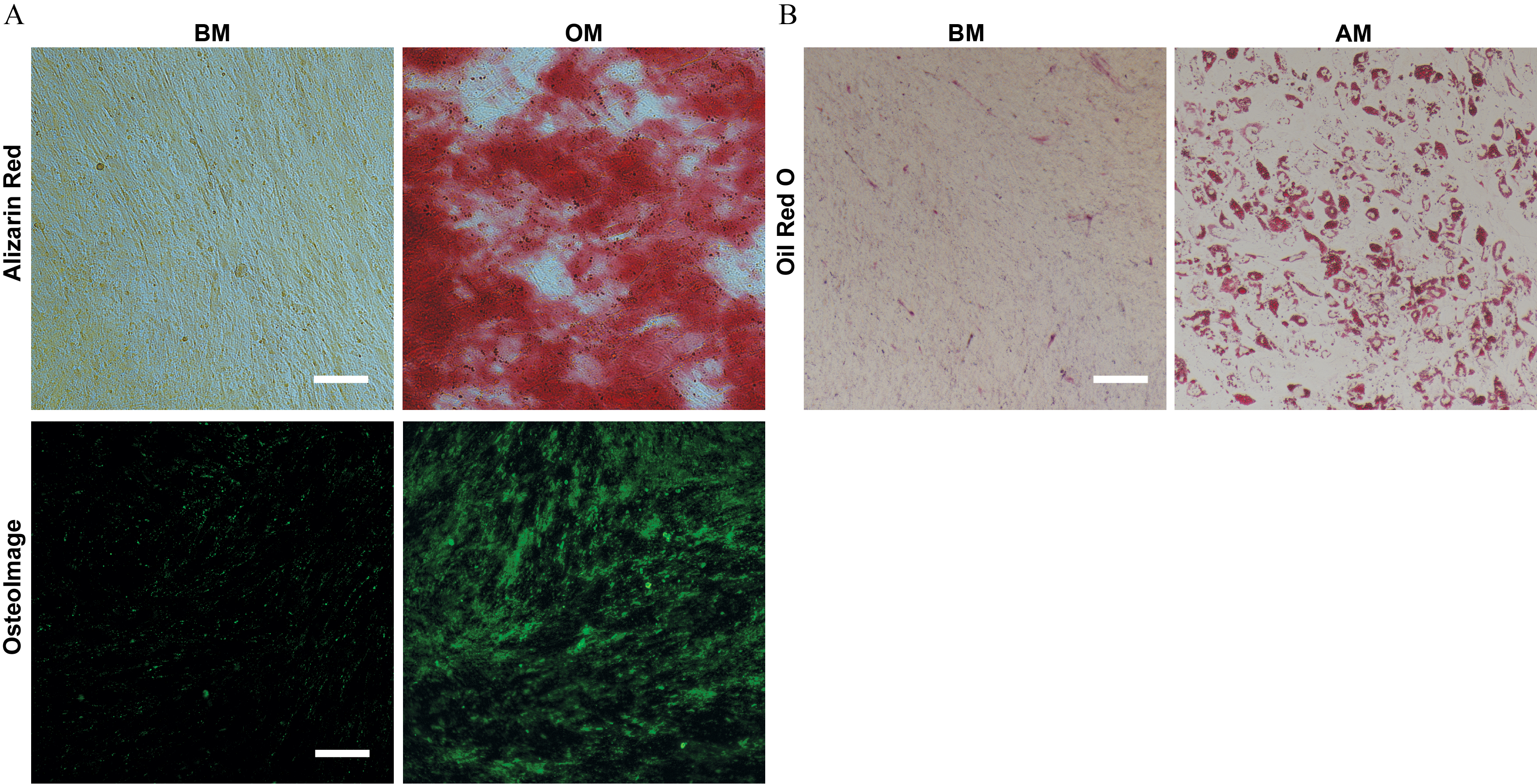


**Figure S2.** **A)** Alizarin Red staining and OsteoImage staining of hMSCs cultured in TCPs for 21 days in BM and in OM, which, compared to controls, indicated increased calcium and HA deposition, respectively. **B)** Oil Red O staining of hMSCs cultured in TCPs for 21 days in BM and in AM demonstrated increased intracellular vesicles compared to a control. Scale bars represent 100 µm.

Since it has been demonstrated that engineered enthesis constructs can be obtained by exposing MSCs to a defined composition of differentiation media,^[30]^ we tested combinations of selective differentiation factors (**Figures S3** and **S4**). For the optimization of the differentiation protocols, hMSCs were first cultured in a collagen-sandwich system established in TCPs. There, the cells were exposed to BM supplemented with Transforming Growth Factor (TGF-)β2 – ‘tendon differentiation medium’ (TDM) – to induce tenogenesis or supplemented with a combination of TGF-β2 and Growth/Differentiation Factor (GDF)5 – ‘fibrocartilage differentiation medium’ (FDM) – to induce fibrochondrogenesis. The enthesis differentiation phenotype when culturing in these differentiation media was assessed by gene expression analysis after 1, 3, 5 and 8 days and by immunocytochemistry at day 5 and compared to cells cultured in BM.

Culturing in TDM in TCPs enhanced the mRNA expression level of scleraxis (*Scx*), a key regulator of tenogenesis,^[31]^ on average 5.02-fold over the time points analyzed compared to BM (Figure S3A), which is in line with previous reports.^[32]^ Although an opposite trend was measured in MSCs cultured on an anisotropic scaffold,^[30b]^ the combinations of TGF-β2 with microtopographies^[33]^ or matrix components^[34]^ have been proven to have a synergistic effect on the induction of *Scx* expression, with collagen substrates inducing higher expression levels compared to other matrix components.^[34]^ The collagen-sandwich culture in our system might be similarly supporting the *Scx* expression of the hMSCs and their differentiation towards the tenogenic lineage. Mohawk (*Mkx*), a regulator of tendon development,^[35]^ was also upregulated at day 3, 5 and 8, in line with a previous study showing upregulation at day 8.^[35]^ Collagen (*Col-*)*I* and *Col-III*, the corresponding proteins being the most abundant extra cellular matrix (ECM) proteins in tendon and ligament,^[36]^ were also upregulated after 8 days of treatment with TGF-β2. The expression levels of the fibrochondrogenic markers SRY-box transcription factor 9 (*Sox9*),^[37]^ *Col-II* and *Col-X* were comparable to those of the controls cultured in BM.

On the other hand, hMSCs cultured in FDM in TCPs enhanced the gene expression level of the *Sox9* similarly after 3 days of culture (Figure S4A). The expression of *Col-II*,^[38]^ the related protein being another ECM component, reached a 4.03-fold increased expression relative to BM after 8 days of culture. The role of GDF5 in promoting hypertrophic chondrocyte-like differentiation has been described in both MSCs derived from healthy donors and OA patients, measured as an enhanced expression of *Sox9* and *Col-II* after 7 days of culture,^[39]^ in line with our observation. The expression level of *Col-III*, the corresponding protein being one of the main components of the fibrocartilage ECM,^[40]^ significantly increased at the time points analyzed compared to BM. *Col-X*, a hypertrophy-related marker,^[41]^ reached a 28.6-fold increased expression relative to BM after 5 days of culture, which remained similar at day 8. Although it has been shown that GDF5 reduces *Col-X* expression levels,^[42]^ another work demonstrated an opposite trend,^[43]^ supporting the key role of GDF5 in chondrogenic differentiation.^[44]^ The tenogenic markers *Scx*, *Mkx* and *Col-I* showed similar expression levels as cells cultured in BM.

Next, we confirmed tenogenic and fibrochondrogenic differentiation in TCPs by analyzing the protein expression levels of COL-III and SCX and of COL-II and SOX9, respectively (Figures S3B and C and S4B and C). Quantitative analysis of the percentage of cell area stained positively for COL-III revealed significantly increased deposition in cells cultured in TDM compared to those cultured in BM. Quantification of SCX mean intensity also showed increased levels in cells cultured in TDM relative to those cultured in BM. On the other hand, incubation in FDM showed an increased deposition of COL-II, measured as percentage of covered cell area, compared to BM. Similarly, a significantly higher number of cell nuclei positive for SOX9 was measured in cells cultured in FDM relative to those cultured in BM.


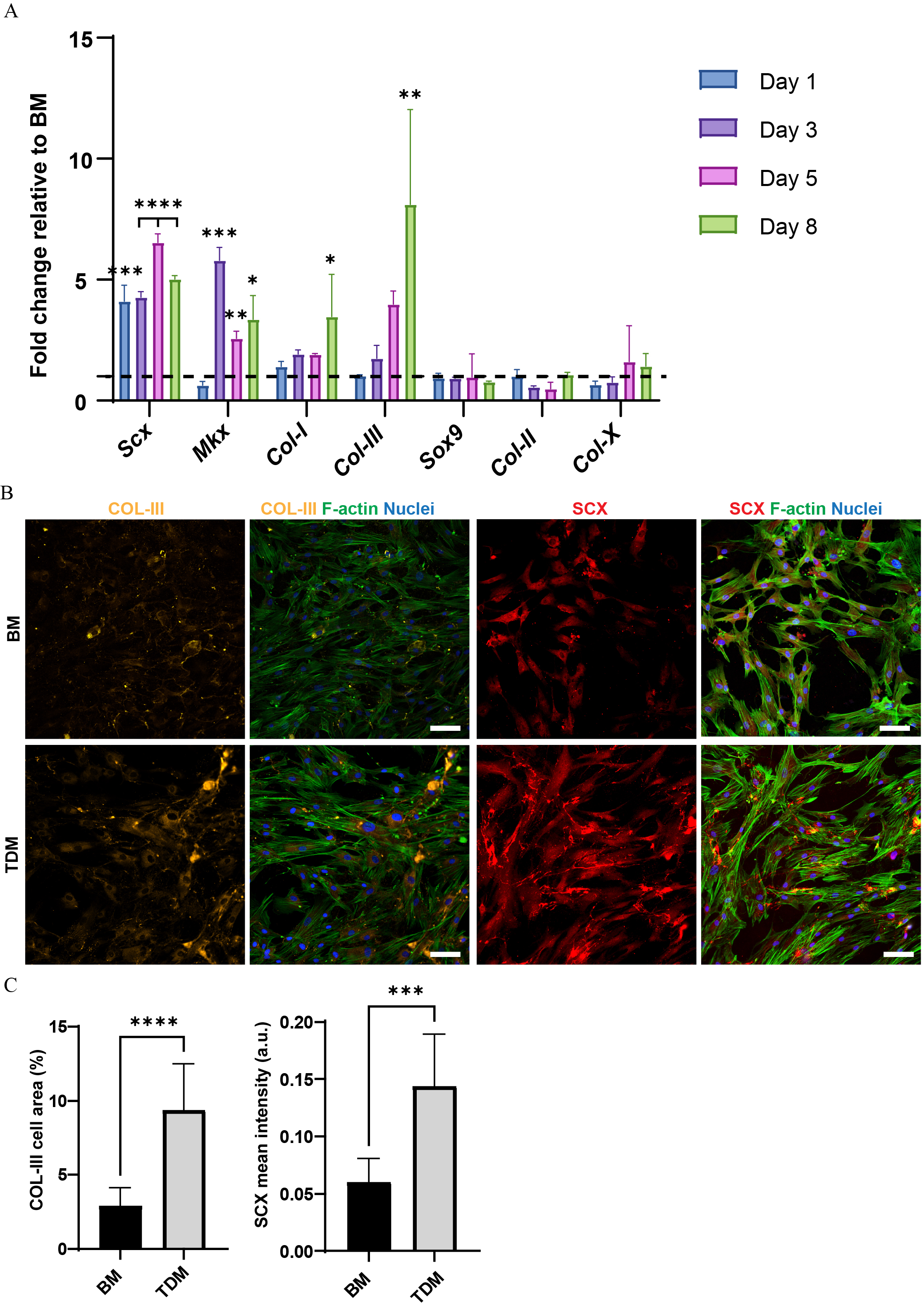


**Figure S3.** **A)** Bar graphs showing the expression of tenogenic and fibrochondrogenic genes in hMSCs cultured in TCPs for 1, 3, 5 and 8 days in TDM. Bars represent mean values and error bars standard deviations. Significance was determined by a two-tailed unpaired Student’s t-test. * p < 0.05, ** p < 0.01, *** p < 0.001 and **** p < 0.0001. N = 3. **B)** Confocal fluorescence images of hMSCs cultured in TCPs for 5 days in BM and in TDM. The cells were immunostained for COL-III (yellow) and SCX (red), and stained with phalloidin for F-actin (green) and with 4’,6-diamidino-2-phenylindole (DAPI) for nuclei (blue). Scale bars represent 100 µm and apply to all images. **C)** Quantification of COL-III production measured as the percentage of covered cell area and quantification of SCX intensity. Bars represent mean values and error bars standard deviations. Significance was determined by a two-tailed unpaired Student’s t-test. *** p < 0.001 and **** p < 0.0001. N = 3.


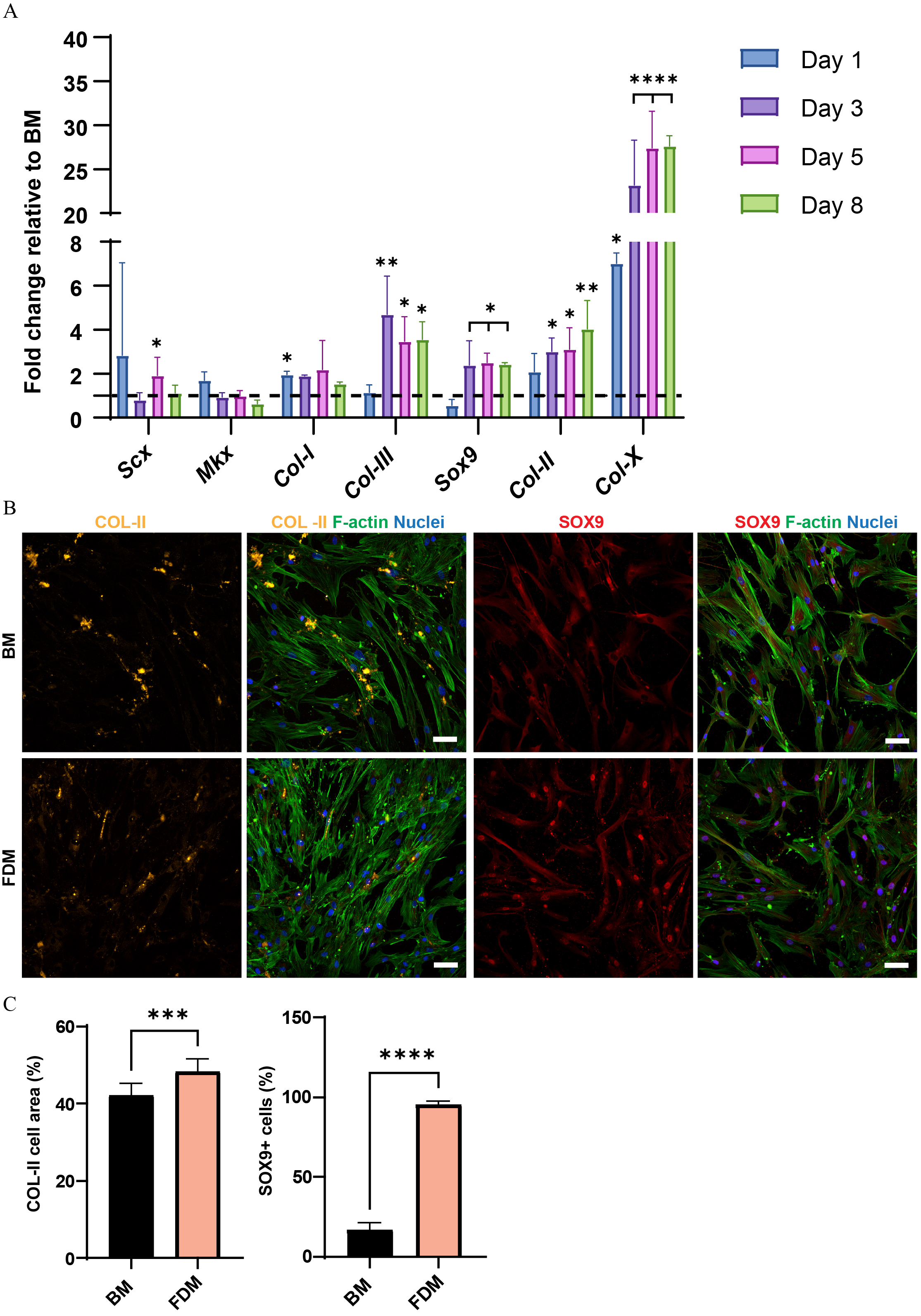


**Figure S4.** **A)** Bar graphs showing the expression of tenogenic and fibrochondrogenic genes in hMSCs cultured in TCPs for 1, 3, 5 and 8 days in FDM. Bars represent mean values and error bars standard deviations. Significance was determined by a two-tailed unpaired Student’s t-test. * p < 0.05, ** p < 0.01, **** p < 0.0001. N = 3. **B)** Confocal fluorescence images of hMSCs cultured in BM and in FDM for 5 days. The cells were immunostained for COL-II (yellow) and SOX9 (red), and stained with phalloidin for F-actin (green) and with DAPI for nuclei (blue). Scale bars represent 100 µm and apply to all images. **C)** Quantification of COL-II production measured as the percentage of covered cell area and quantification of the percentage of SOX9-positive cells. Bars represent mean values and error bars standard deviations. Significance was determined by a two-tailed unpaired Student’s t-test. *** p < 0.001 and **** p < 0.0001. N = 3.


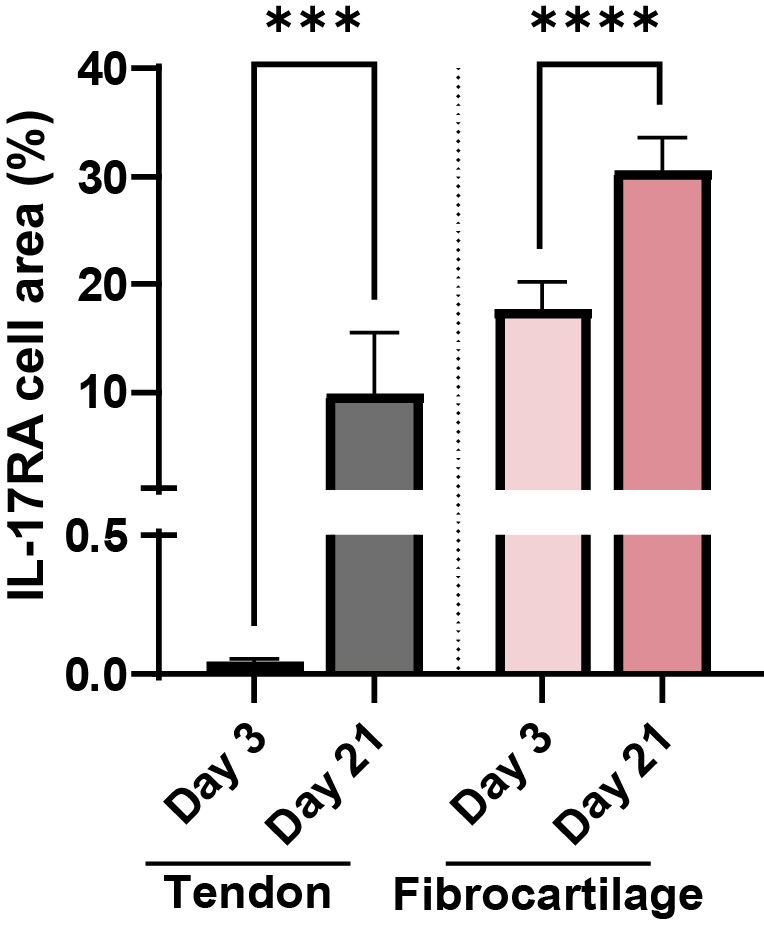


**Figure S5.** Bar graph showing the percentage of cell area stained for IL-17RA at day 3 (acute inflammation) and day 21 (chronic inflammation) in the tendon and fibrocartilage compartment of the enthesis-on-chip device. Bars represent mean values and error bars standard deviations. Significance was determined by a two-tailed unpaired Student’s t-test *** p < 0.001 and **** p < 0.0001. N = 3.


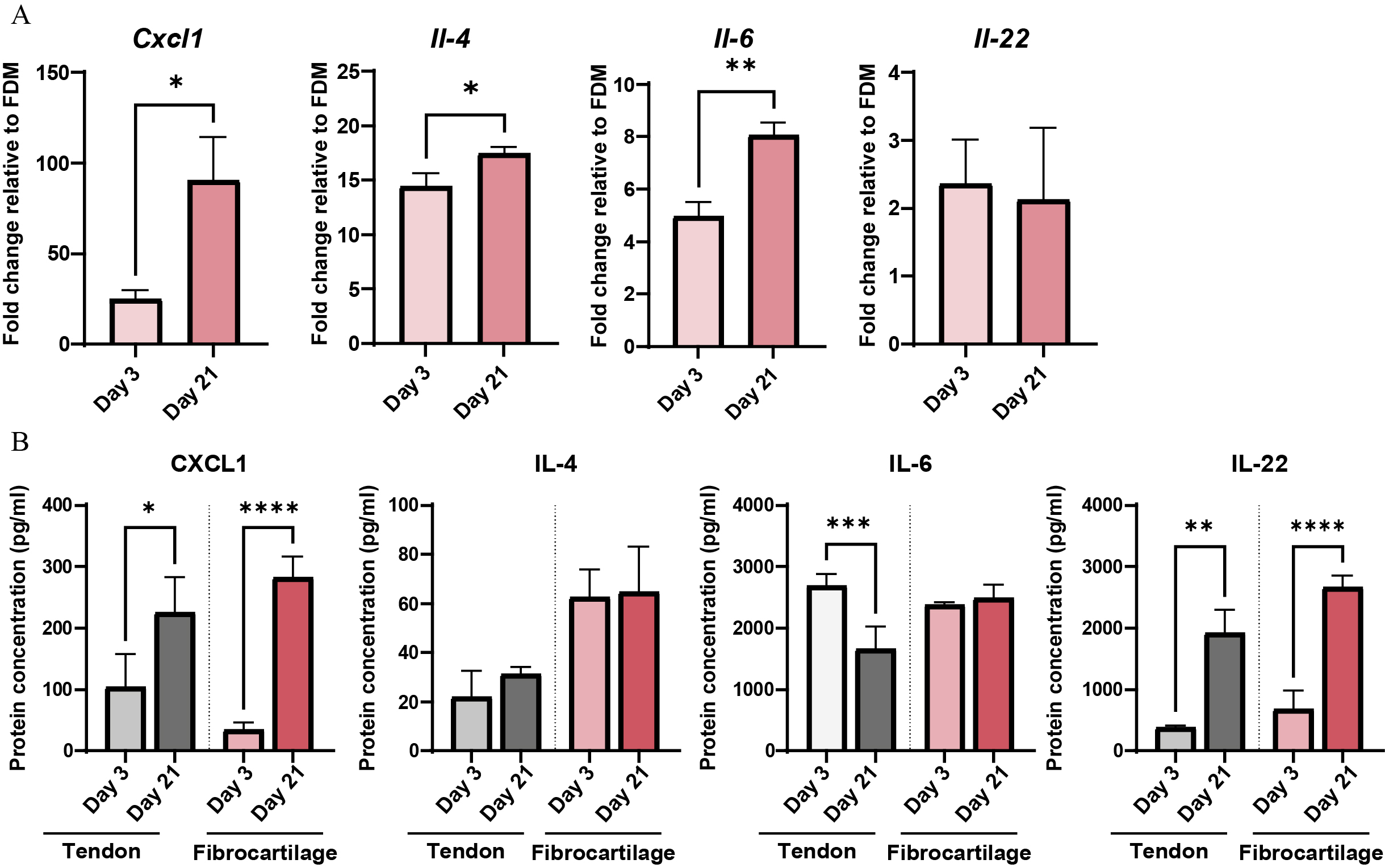


**Figure S6.** **A)** Bar graphs showing the expression levels of inflammation-related genes at day 3 (acute inflammation) and day 21 (chronic inflammation) in the fibrocartilage compartment. Bars represent mean values and error bars standard deviations. Significance was determined by a two-tailed unpaired Student’s t-test. * p < 0.05 and ** p < 0.01. N = 3. **B)** Bar graphs showing the multiplex analysis of the released levels of inflammatory protein measured at day 3 (acute inflammation) and day 21 (chronic inflammation) in the tendon and fibrocartilage compartment. Bars represent mean values and error bars standard deviations. Significance was determined by a two-tailed unpaired Student’s t-test. * p < 0.05, ** p < 0.01, *** p < 0.001 and **** p < 0.0001. N = 3.


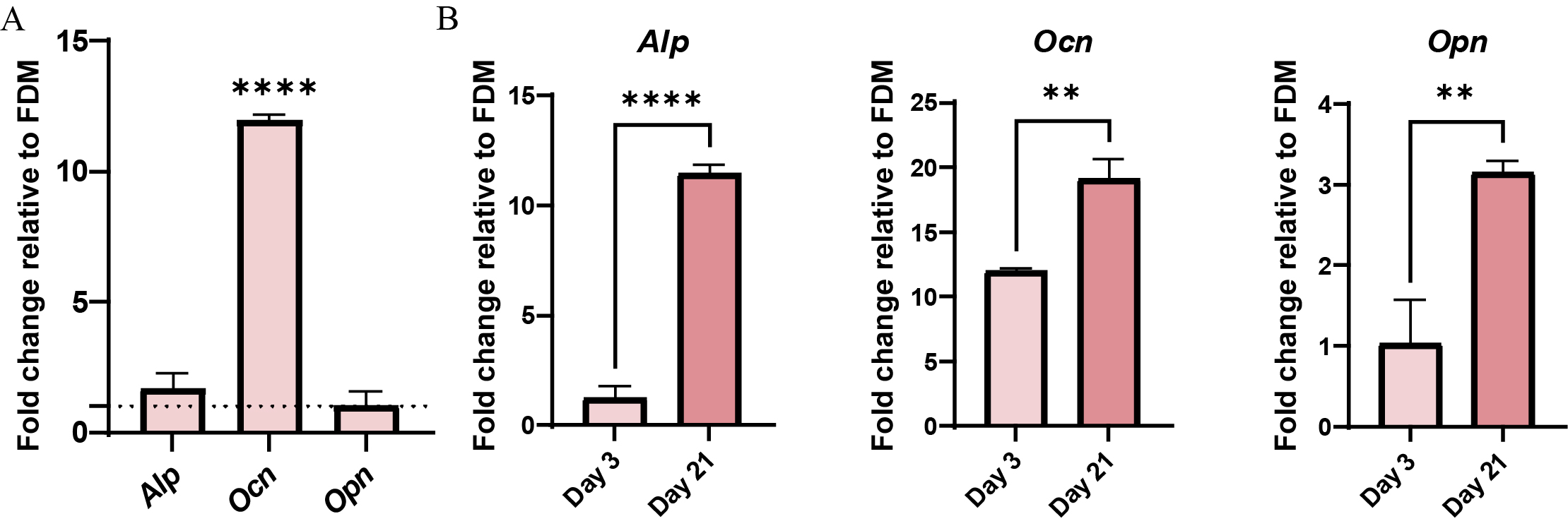


**Figure S7.** **A)** Bar graph showing the expression of osteogenic genes in the fibrocartilage compartment after the end of the induction of acute inflammation. Bars represent mean values and error bars standard deviations. Significance was determined by a two-tailed Student’s unpaired t-test. **** p < 0.0001. N = 3. **B)** Bar graphs showing the expression levels of osteogenic genes at day 3 (acute inflammation) and day 21 (chronic inflammation) in the fibrocartilage compartment. Bars represent mean values and error bars standard deviations. Significance was determined by a two-tailed unpaired Student’s t-test. ** p < 0.01 and **** p < 0.0001. N = 3.


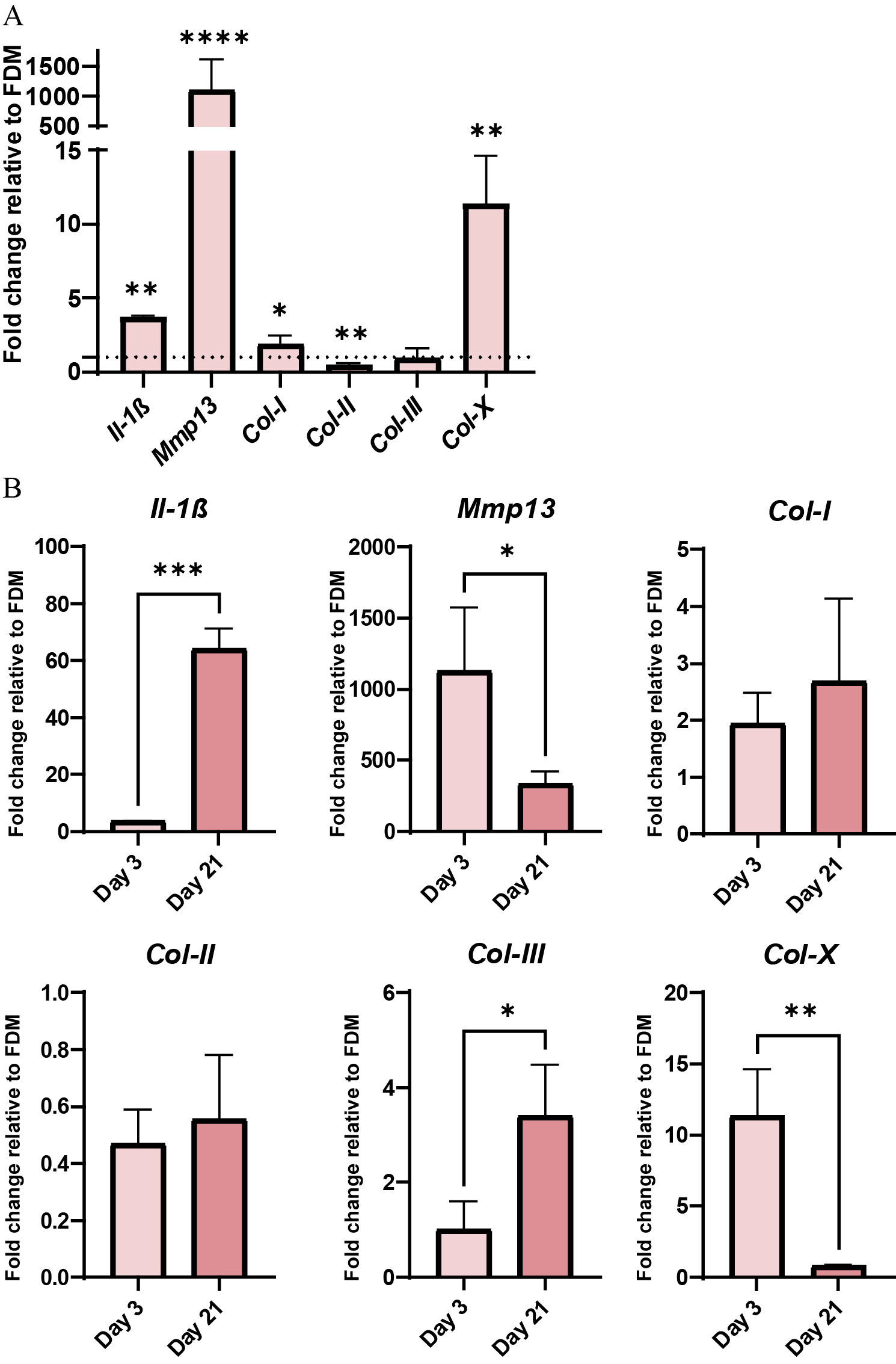


**Figure S8.** **A)** Bar graph showing the expression of ECM-related genes in the fibrocartilage compartment after the end of the induction of acute inflammation. Bars represent mean values and error bars standard deviations. Significance was determined by a two-tailed Student’s unpaired t-test. ** p < 0.01 and **** p < 0.0001. N = 3. **B)** Bar graphs showing the expression levels of ECM-related genes at day 3 (acute inflammation) and day 21 (chronic inflammation) in the fibrocartilage compartment. Bars represent mean values and error bars standard deviations. Significance was determined by a two-tailed unpaired Student’s t-test. * p < 0.05, ** p < 0.01 and *** p < 0.001. N = 3.

**Table S1. Primer sequences used for RT-qPCR.**

| **Gene** | **Forward Primer** | **Reverse Primer** |
| --- | --- | --- |
| *Scx* | CAGCCCAAACAGATCTGCACCTT | CTGTCTTTCTGTCGCGGTCCTT |
| *Mkx* | TGGTTTGCTAATGCAAGACG | CCTTCGTTCATGTGGGTTCT |
| *Col-I* | AGCGGACGCTAACCCCCTC | CAGACGGGACAGCACTCGCC |
| *Col-III* | TACTTCTCGCTCTGCTTCATCC | GAACGGATCCTGAGTCACAGAC |
| *Sox9* | GGCGGAGGAAGTCGGTGAAGAA | GCTCATGCCGGAGGAGGAGTGT |
| *Col-II* | AACCAGATTGAGAGCATCCG | ACCTTCATGGCGTCCAAG |
| *Col-X* | CCCTCTTGTTAGTGCCAACC | AGATTCCAGTCCTTGGGTCA |
| *Cxcl1* | AGTGGCACTGCTGCTCCT | TGGATGTTCTTGGGGTGAAT |
| *Il-4* | CCGTAACAGACATCTTTGCTGCC | GAGTGTCCTTCTCATGGTGGCT |
| *Il-6* | ACTCACCTCTTCAGAACGAATTG | CCATCTTTGGAAGGTTCAGGTTG |
| *Il-22* | CTCCACAGCGGCATAGCCT | ACATGCAGCTTCCAGCTGG |
| *Alp* | ATCTTTGGTCTGGCCCCCATG | AGTCCACCATGGAGACATTCTCTC |
| *Ocn* | TCACACTCCTCGCCCTATTG | GAAGAGGAAAGAAGGGTGCC |
| *Opn* | TCACCAGTCTGATGAGTCTCACCATTC | TAGCATCAGGGTACTGGATGTCAGGT |
| *Il-1β* | ATGATGGCTTATTACAGTGGCAA | GTCGGAGATTCGTAGCTGGA |
| *Mmp13* | ATGCAGTCTTTCTTCGGCTTAG | ATGCCATCGTGAAGTCTGGT |
| *Gapdh* | GGAGCGAGATCCCTCCAAAAT | GGCTGTTGTCATACTTCTCATGG |
| *18S rRNA* | GTAACCCGTTGAACCCCATT | CCATCCAATCGGTAGTAGCG |

**Table S2. Primary antibodies used in the study.**

| **Primary Antibody** | **Compartment** | **Dilution** | **Supplier** | **Catalogue Number** |
| --- | --- | --- | --- | --- |
| Rabbit anti-collagen-III | Top | 1:100 | Abcam | ab7778 |
| Rabbit anti-scleraxis | Top | 1:200 | Abcam | ab58655 |
| Mouse anti-collagen-II | Bottom | 1:100 | Abcam | ab185430 |
| Mouse anti-SOX9 | Bottom | 1:100 | Abcam | ab76997 |
| Rabbit anti-IL-17RA | Top and bottom | 1:50 | Abcam | ab180904 |

**Table S3. Secondary antibodies used in the study.**

| **Primary Antibody** | **Secondary Antibody** | **Dilution** | **Supplier** |
| --- | --- | --- | --- |
| Rabbit anti-collagen-III | Alexa Fluor 568  goat anti-rabbit | 1:1000 | Thermo Fisher |
| Rabbit anti-scleraxis | Alexa Fluor 647  goat anti-rabbit | 1:500 | Thermo Fisher |
| Mouse anti-collagen-II | Alexa Fluor 568  goat anti-mouse | 1:1000 | Thermo Fisher |
| Mouse anti-SOX9 | Alexa Fluor 647  goat anti-mouse | 1:1000 | Thermo Fisher |
| Rabbit anti-IL-17RA | Alexa Fluor 647  goat anti-rabbit | 1:500 | Thermo Fisher |

**References**

[30] a)S. Font Tellado, W. Bonani, E. R. Balmayor, P. Foehr, A. Motta, C. Migliaresi, M. van Griensven, *Tissue Eng Part A* **2017**, 23, 859; b)S. Font Tellado, S. Chiera, W. Bonani, P. S. P. Poh, C. Migliaresi, A. Motta, E. R. Balmayor, M. van Griensven, *Acta Biomater* **2018**, 72, 150; c)R. Gottardi, K. Moeller, R. Di Gesù, R. S. Tuan, M. van Griensven, E. R. Balmayor, *Front Mater* **2021**, 8.

[31] a)P. Alberton, C. Popov, M. Prägert, J. Kohler, C. Shukunami, M. Schieker, D. Docheva, *Stem Cells Dev* **2012**, 21, 846; b)H. Liu, S. Zhu, C. Zhang, P. Lu, J. Hu, Z. Yin, Y. Ma, X. Chen, H. OuYang, *Cell Tissue Res* **2014**, 356, 287.

[32] S. K. Theodossiou, J. Tokle, N. R. Schiele, *Biochem Biophys Res Commun* **2019**, 508, 889.

[33] S. Vermeulen, N. Roumans, F. Honig, A. Carlier, D. G. A. J. Hebels, A. Dede Eren, P. T. Dijke, A. Vasilevich, J. de Boer, *Biomaterials* **2020**, 259, 120331.

[34] R. I. Sharma, J. G. Snedeker, *Biomaterials* **2010**, 31, 7695.

[35] G. Chen, D. Fan, W. Zhang, S. Wang, J. Gu, Y. Gao, L. He, W. Li, C. Zhang, M. Li, Y. Zhang, Z. Liu, Q. Hao, *Stem Cell Res Ther* **2021**, 12, 426.

[36] D. Stanco, C. Caprara, G. Ciardelli, L. Mariotta, M. Gola, G. Minonzio, G. Soldati, *PLoS One* **2019**, 14, e0212192.

[37] S. Thomopoulos, R. Das, V. Birman, L. Smith, K. Ku, E. L. Elson, K. M. Pryse, J. P. Marquez, G. M. Genin, *Tissue Eng Part A* **2011**, 17, 1039.

[38] W. Zhong, W. Zhang, S. Wang, J. Qin, *PLoS One* **2013**, 8, e61283.

[39] M. Ratnayake, M. Tselepi, R. Bloxham, F. Plöger, L. N. Reynard, J. Loughlin, *PLoS One* **2017**, 12, e0176523.

[40] S. Hosseininia, M. A. Weis, J. Rai, L. Kim, S. Funk, L. E. Dahlberg, D. R. Eyre, *Osteoarthritis Cartilage* **2016**, 24, 1029.

[41] C. A. Knuth, E. Andres Sastre, N. B. Fahy, J. Witte-Bouma, Y. Ridwan, E. M. Strabbing, M. J. Koudstaal, J. van de Peppel, E. B. Wolvius, R. Narcisi, E. Farrell, *Eur Cell Mater* **2019**, 38, 106.

[42] B. I. Ayerst, R. A. Smith, V. Nurcombe, A. J. Day, C. L. Merry, S. M. Cool, *Tissue Eng Part A* **2017**, 23, 275.

[43] Y. Hatakeyama, R. S. Tuan, L. Shum, *J Cell Biochem* **2004**, 91, 1204.

[44] A. D. Mazzocca, D. Chowaniec, M. B. McCarthy, K. Beitzel, M. P. Cote, W. McKinnon, R. Arciero, *Knee Surg Sports Traumatol Arthrosc* **2012**, 20, 1666.
